# Understanding paralogous epilepsy–associated GABA_A_ receptor variants: Clinical implications, mechanisms, and potential pitfalls

**DOI:** 10.1073/pnas.2413011121

**Published:** 2024-12-06

**Authors:** Anthony S. H. Kan, Ali S. Kusay, Nazanin A. Mohammadi, Susan X. N. Lin, Vivian W. Y. Liao, Gaetan Lesca, Sabrine Souci, Mathieu Milh, Palle Christophersen, Mary Chebib, Rikke S. Møller, Nathan L. Absalom, Anders A. Jensen, Philip K. Ahring

**Affiliations:** ^a^School of Medical Sciences, Faculty of Medicine and Health, Brain and Mind Centre, The University of Sydney, Sydney, NSW 2006, Australia; ^b^Department of Biomedical and Clinical Sciences, Linköping University, Linköping SE-581 83, Sweden; ^c^Department of Epilepsy Genetics and Personalized Treatment, Danish Epilepsy Center Filadelfia, Member of the European Reference Network EpiCARE, Dianalund DK-4293, Denmark; ^d^Department of Regional Health Research, University of Southern Denmark, Odense DK-5230, Denmark; ^e^Department of Medical Genetics, Member of the European Reference Network EpiCARE, Hospices Civils de Lyon, Lyon 69002, France; ^f^Institut Neuromyogène, CNRS UMR 5310–INSERM U1217, Université de Lyon, Université Claude Bernard Lyon 1, Lyon 69008, France; ^g^Department of Neurology, Hospices Civils de Lyon, Lyon Sud University Hospital, Pierre Bénite 69495, France; ^h^Department of Pediatric Neurology, Assistance Publique – Hôpitaux de Marseille, La Timone Children’s Hospital, Marseille 13005, France; ^i^Institut de Neurobiologie de la Méditerranée, INSERM, Aix-Marseille Université, Marseille 13273, France; ^j^Saniona A/S, Ballerup DK-2750, Denmark; ^k^School of Science, Western Sydney University, Penrith, NSW 2751, Australia; ^l^Department of Drug Design and Pharmacology, Faculty of Health and Medical Sciences, University of Copenhagen, Copenhagen DK-2100, Denmark

**Keywords:** neurodevelopmental disorders, epilepsy, GABRA1, GABRB3, GABRG2

## Abstract

Recent discoveries have revealed that genetic variants in γ-aminobutyric acid type A (GABA_A_) receptor subunits can lead to both gain-of-function (GOF) and loss-of-function (LOF) receptors. GABA_A_ receptors, however, have a pseudosymmetrical pentameric assembly, and curiously diverse functional outcomes have been reported for certain homologous variants in paralogous genes (paralogous variants). To investigate this, we assembled a cohort of 11 individuals harboring paralogous M1 proline missense variants in *GABRA1*, *GABRB2*, *GABRB3,* and *GABRG2.* Seven mutations (α1^P260L^, α1^P260S^, β2^P252L^, β3^P253L^, β3^P253S^, γ2^P282A^, and γ2^P282S^) in α1β2/3γ2 receptors were analyzed using electrophysiological examinations and molecular dynamics simulations. All individuals in the cohort were diagnosed with developmental and epileptic encephalopathy, with a median seizure onset age of 3.5 mo, and all exhibited global developmental delay. The clinical data for this cohort aligned with established GABA_A_ receptor GOF but not LOF cohorts. Electrophysiological assessments revealed that all variants caused GOF by increasing GABA sensitivity by 3- to 23-fold. In some cases, this was accompanied by LOF traits such as reduced maximal current amplitude and enhanced receptor desensitization. The specific subunit mutated and whether the mutation occurred in one or two subunits within the pentamer influenced the overall effects. Molecular dynamics simulations confirmed similar structural changes from all mutations, but with position-dependent asymmetry. These findings establish that paralogous variants affecting the 100% conserved proline residue in the M1 transmembrane helix of GABA_A_R subunits all lead to overall GOF traits. The unexpected asymmetric and mixed effects on receptor function have broader implications for interpreting functional analyses for multimeric ion-channel proteins.

## Significance

Pathogenic variants in γ-aminobutyric acid type A (GABA_A_)-receptor subunits are associated with severe developmental and epileptic encephalopathies. For the advancement of precision medicine, understanding both the pathogenicity and functional consequences of novel variants is crucial. To investigate whether insights from paralogous genes can serve as predictors, we studied a cohort of eleven individuals harboring paralogous missense variants in a conserved proline residue within the first transmembrane helix of GABA_A_ receptor subunits. Despite diverse functional outcomes, all variants led to overall gain-of-function with increased GABA sensitivity being key to the clinical phenotype. Our findings support the use of information from paralogous variants in certain cases, but also highlight the importance of detailed functional characterization when assessing the molecular phenotypes of variants.

**Fig. 1.**
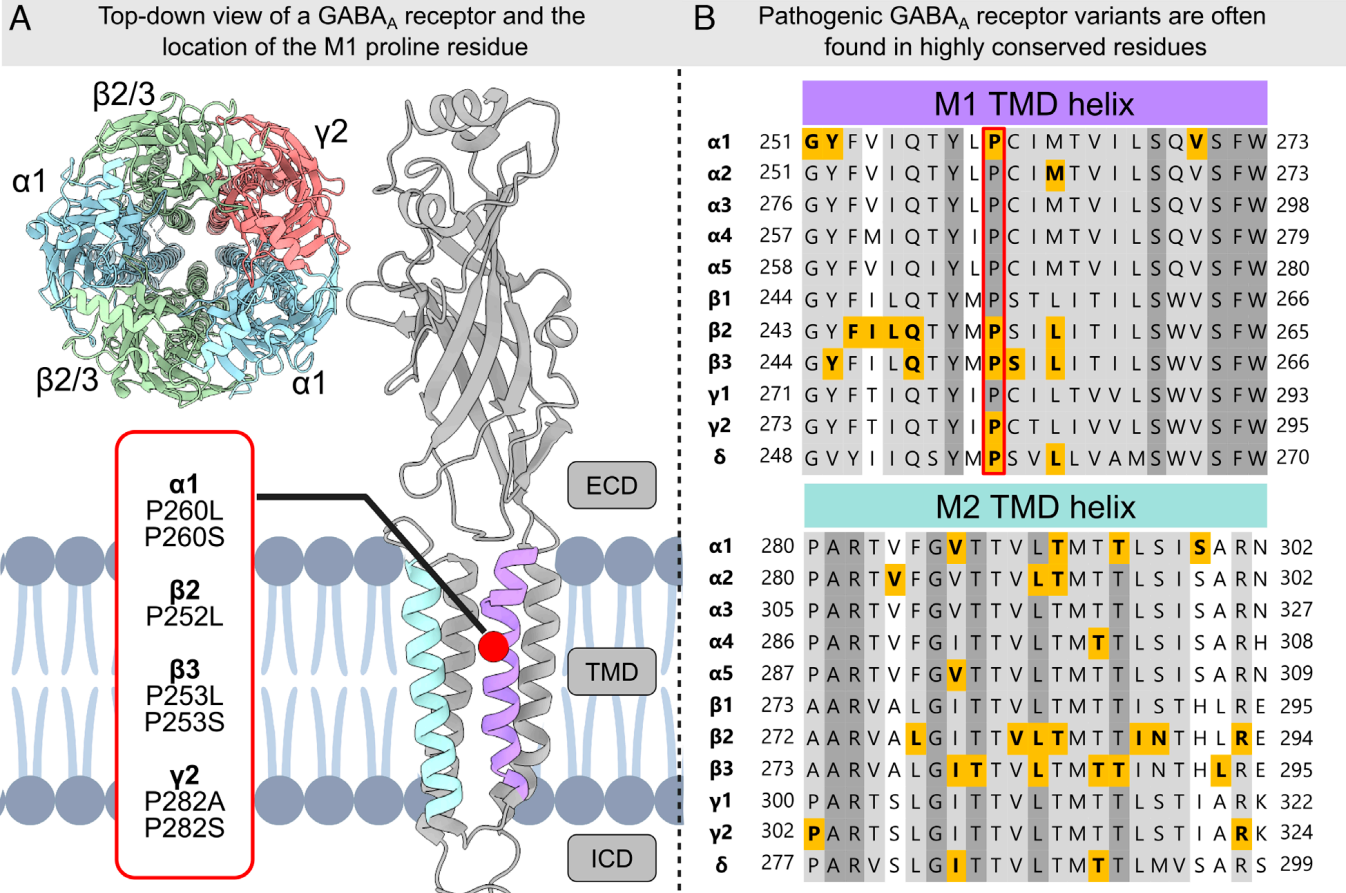
The M1 proline residue is fully conserved in GABA_A_R subunits. (*A*) *Top*-*Left*: Cryo-EM structure of a pentameric GABA_A_R viewed from the extracellular side (PDB: 6HUP). *Right*: Cryo-EM structure of an α1 subunit viewed along the plasma membrane illustrating the M1 (purple) and M2 (teal) transmembrane domain (TMD) helices, and the position of the conserved M1 proline (red) and presumed pathogenic variants. ECD: extracellular domain; ICD: intracellular domain. (*B*) Amino acid sequence alignment of the M1 and M2 TMD helixes of α(1-5), β(1-3), γ(1-2), and δ subunits with the conserved proline bracketed in red. Based on a literature review, variants with published functional data are highlighted bold in yellow, with most of them located in fully (dark gray) or highly (light gray) conserved residues (2–16).

γ-aminobutyric acid type A receptors (GABA_A_Rs) are members of the Cys-loop ligand-gated ion channel superfamily and comprise five subunits arranged in a pseudosymmetrical arrangement. The archetypical GABA_A_R subtypes consist of two α subunits, two β subunits and a single γ or δ subunit in a counterclockwise γ/δ-β-α-β-α arrangement (Fig. 1*A*) (1). Receptor subtypes can be assembled from six α (α1 to 6), three β (β1 to 3), three γ (γ1 to 3) subunits, and one δ subunit encoded by the genes *GABRA1-6*, *GABRB1-3*, *GABRG1-3,* and *GABRD*, respectively.

Apart from *GABRA6* and *GABRG3*, all major GABA_A_R genes have been clearly linked to neurodevelopmental disorders and epilepsy (17, 18). Genetic variants in GABA_A_R subunits are for example frequently observed in individuals with developmental and epileptic encephalopathies (DEE) (2). DEE encompasses a group of severe epilepsies characterized by seizures, interictal epileptiform activity on EEG, and encephalopathy, which involves significant developmental delay (DD) and cognitive impairment (19). Furthermore, recent studies have established clear correlations between functional and clinical outcomes for these variants (3–5). For example, individuals with gain-of-function (GOF) variants typically present with more severe clinical phenotypes than those with loss-of-function (LOF) variants. Importantly, the functional outcome of a variant may have implications for whether antiseizure medications that enhance GABAergic neurotransmission should be used or avoided (6). Moreover, the recent approval of antisense oligonucleotides (ASOs) for certain rare diseases, such as spinal muscular atrophy, marks an interesting new advancement. ASOs, tailored to up-regulate or down-regulate the expression of mutated GABA_A_R subunit mRNAs, could potentially present disease-modifying interventions. Hence, regardless of the intervention type, it is crucial to know whether variants are GOF or LOF, yet such information is rarely available for newly identified variants.

GABA_A_R subunits exhibit significant overall sequence identity, ranging from 34 to 87%, and novel variants typically appear in conserved positions, where the amino acid identity can be up to 100% across all subunits. For instance, pathogenic variants are enriched at transmembrane helices (M1-M4), which form the receptor’s ion channel pore (1). Yet, even in these highly conserved regions, functional information is only available for a limited number of variants, scattered among the different subunits (Fig. 1*B*). Therefore, exploring alternative avenues to gather information regarding functional outcomes is essential. Within the ion channel field, it is generally assumed that variants at identical amino acid positions in closely related genes will likely yield similar functional effects. In fact, pathogenicity prediction databases incorporate information of homologous variants in paralogous genes (hereafter termed paralogous variants), when functional data for a specific variant are unavailable [e.g., the AlphaMissense prediction tool (20)]. This approach has successfully been used to predict functional changes at sodium and calcium channels (21, 22). Based on such success, it has even been suggested recently that paralogous variant information could be used for applying the ACMG PS1/PM5 criteria to novel variants (23).

Unlike sodium or potassium channels and other gene families, the individual subunits in GABA_A_Rs are not functionally equivalent. Instead, the pseudosymmetrical receptor structure has evolved to include subunit-specific functional regions with distinct roles in channel function. This is evident in the extracellular region, where GABA-binding sites are located on different sides of the α and β subunits. In contrast, the transmembrane pore-forming regions are expected to display higher functional equivalence across subunit types. Therefore, it is most meaningful to compare variants in regions of high functional equivalence. Some studies support the notion that paralogous variants in transmembrane regions can have similar functional effects. For instance, pathogenic variants affecting a fully conserved threonine residue in the M2 helix of *GABRA4*, *GABRB3*, and *GABRD* all result in a GOF molecular phenotype (5, 7, 8). However, observations for a similarly conserved proline residue in the center of the M1 transmembrane helix are conflicting (Fig. 1 *A* and *B*). Variants of this M1 proline have been identified across all major subunit types (8–11, 24–28), yet functional analyses revealed surprisingly diverse outcomes spanning from LOF traits [*GABRA1*, *GABRB2,* and *GABRG2* variants (9–11)] over limited functional changes [*GABRB3* variant (5)] to GOF traits [*GABRD* (8)]. Thus, a clear understanding of the utility of paralogous variants in GABA_A_Rs remains elusive.

The proposed use of information from paralogous variants to apply the ACMG criteria highlights the need to assess whether functional data supports such use, and whether the literature provides dependable sources for functional outcomes. Given the pseudosymmetrical nature of GABA_A_R assembly, variants in the M1 proline are particularly suitable for this study. These variants exist in many different subunit types and the reported functional data for them are conflicting. Therefore, we tested the hypothesis that paralogous variants at the M1 proline of subunits that assemble into the major synaptic α1β2/3γ2 GABA_A_R subtypes lead to similar functional outcomes and comparable clinical phenotypes.

## Results

**Fig. 2.**
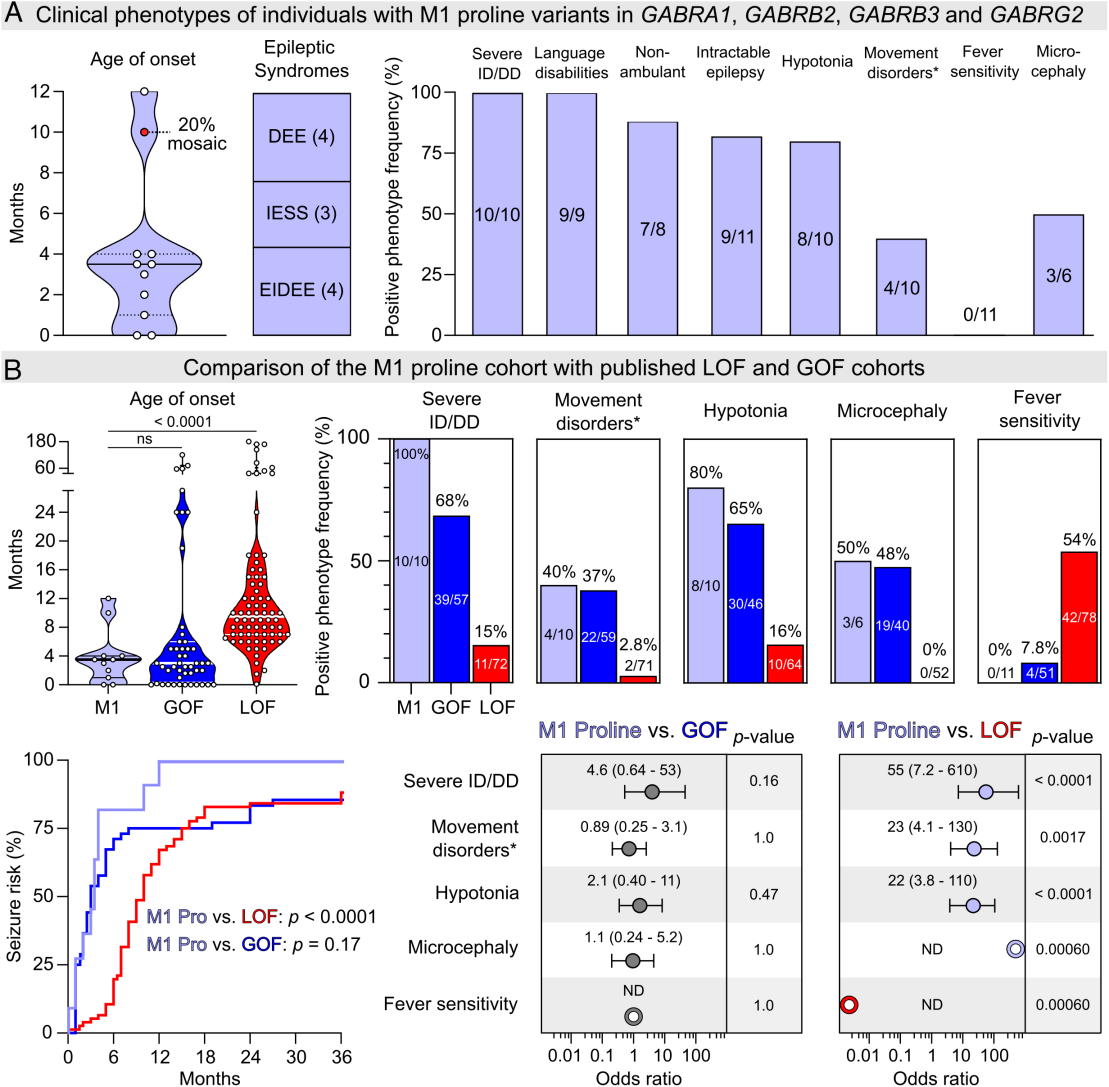
Clinical features of individuals with M1 proline variants in *GABRA1*, *GABRB2*, *GABRB3,* and *GABRG2*. (*A*) Distribution of age of seizure onset, epileptic syndromes, and eight key clinical features in the M1 proline cohort are shown. Solid line and dotted lines in the violin plot depict the median and interquartile range, respectively. EIDEE: Early infantile developmental and epileptic encephalopathy; IESS: Infantile epileptic spasms syndrome; DEE: Developmental and epileptic encephalopathy. Movement disorders* include dystonia, dyskinesia, choreoathetosis, and athetosis. For detailed information, see Dataset S1. (*B*) Age of seizure onset of the M1 proline cohort (n = 11) was compared against the published LOF (n = 74) and GOF (n = 48) cohorts from *GABRA1/B2/B3* (3–5). The age of onset in the M1 proline cohort was lower than the LOF cohort (Mann–Whitney test, *P* < 0.0001) but statistically insignificant to the GOF cohort (*P* = 0.87). Survival analysis also showed that the M1 proline cohort had a significantly higher seizure risk earlier in life than the LOF cohort (Mantel-Cox test, *P* < 0.0001), but no different to the GOF cohort (*P* = 0.17). Frequency comparisons of five clinical features showed that both M1 and GOF cohorts share similar phenotypic frequencies which differ significantly from those observed in the LOF cohort. OR comparisons were subsequently performed. ORs were displayed along with 95% CI, and statistical significance was determined using two-tailed Fisher’s exact test. OR could not be determined (ND) when either the lower or upper limit of the OR was 0 or infinite, respectively. Lilac circles indicate that the clinical feature is more prevalent in the M1 proline cohort, and red indicates that it is more prevalent in the LOF cohort. Gray indicates no difference in prevalence between the cohorts.

We collected a cohort of 11 individuals with DEE due to seven different de novo M1 proline variants in *GABRA1, GABRB2, GABRB3,* or *GABRG2* encoding the α1, β2, β3, or γ2 subunits, respectively (Fig. 1*A*). Summaries of electroclinical data are presented in Fig. 2 and *SI Appendix*, Fig. S1, while full information is available in Dataset S1.

### *GABRA1* Variants.

Four individuals harbored a M1 proline variant in *GABRA1* with two individuals each for the P260L and P260S variants. All four were diagnosed with either early infantile developmental and epileptic encephalopathy (EIDEE, 1/4) or infantile epileptic spasms syndrome (IESS, 3/4) between the age of 1 and 3.5 mo. Generally, EIDEE is characterized by frequent drug-resistant seizures that begin within the first 3 mo of age, accompanied by abnormal interictal EEG and neurological examination. IESS is another epilepsy syndrome defined by the characteristic seizure type, epileptic spasms, with an onset between 1 and 24 mo of age. For individuals with *GABRA1* variants, the most frequent seizure types included tonic seizures, epileptic spams, focal, myoclonic, atonic, or bilateral to tonic-clonic (TC) seizures. All four individuals had global DD and severe intellectual disability (ID), and the three for whom data was available were nonverbal and nonambulant. Additional features included hypotonia (2/3), microcephaly (1/2), stereotypical hand wringing (1/4), and cortical vision impairment (1/4).

### *GABRB2* and *GABRB3* Variants.

Five individuals harbored a M1 proline variant in the *GABRB2* or *GABRB3* gene. One individual carried the *GABRB2* P252L variant, one the *GABRB3* P253L variant, and three the *GABRB3* P253S variant. All five had global DD, severe ID, and presented with a severe EIDEE (2/5) or DEE (3/5) between day 1 and 10 mo of life. The most frequent seizure types included tonic, focal, and atonic seizures as well as bilateral to TC seizures. All individuals were nonverbal and, with one exception, nonambulant. Individual #6, who was mosaic for the variant (20%), was nonverbal but gained the ability to walk, although he was using a wheelchair due to frequent falls. Movement disorders including dystonia, dyskinesia, chorea, or hyperkinesia were observed in over half the individuals (3/5). Additional features included hypotonia (4/5), microcephaly (2/3), feeding difficulties (2/5), and cortical vision impairment (2/5). One individual died at the age of 6 y due to sudden unexpected death in epilepsy (SUDEP).

### *GABRG2* Variants.

The remaining two individuals carried a M1 proline variant in *GABRG2*, one the P282A and the other the P282S variant. They presented with an EIDEE or DEE at the first day of life or 12 mo of age, respectively. Both had multiple seizure types including focal and myoclonic seizures, atypical absences, trembling, and bilateral to TC seizures. Both individuals had global DD, severe ID, and were nonverbal and nonambulant. Additional features included hypotonia (2/2), dystonia (1/2), roving eye movements/nystagmus (2/2), and strabismus (1/2). One of them died at the age of 18 y due to respiratory failure.

### Summarized Observations for the Cohort.

The 11 individuals in the M1 proline cohort are reported with an overall similar clinical phenotype. The median age of seizure onset for the M1 proline cohort was 3.5 mo (range 1 d to 12 mo) with 9/11 individuals developing seizures before their 4th month of age (Fig. 2*A*). All individuals were severely affected with syndrome classifications of EIDEE, IESS, and DEE. All had global DD, severe ID, were nonverbal and, except for the mosaic individual, nonambulant. Nine out of the 11 individuals had intractable seizures despite the use of a variety of antiseizure medications. Other prominent features included hypotonia and severe movement disorders, while none were reported with fever sensitivity as a triggering factor. Thus, with the current cohort size and data availability, it is not possible to distinguish the clinical outcomes based on which subunit type harbors the variant.

### Comparison of the M1 Proline Cohort with Established GABA_A_R Cohorts.

To determine whether the clinical phenotypes in the M1 proline cohort best reflect GABA_A_R LOF or GOF, the data were compared to previously published *GABRA1, GABRB2,* and *GABRB3* cohorts. Only larger cohorts that functionally establish both LOF and GOF variants were included in this comparison. The largest *GABRA1* cohort comprises 20 individuals with LOF variants and four individuals with GOF variants (3). The largest *GABRB2* cohort includes 13 individuals with LOF and 27 individuals with GOF variants (4). Finally, the largest *GABRB3* cohort includes 47 individuals with LOF and 27 individuals with GOF (5). Since the M1 proline cohort contains individuals with variants in four different subunit types and given that α1 and β2/ β3 subunits are known to assemble in the same receptor complexes, a meaningful method for comparison was to combine the published cohorts. This resulted in combined groups containing 80 individuals with LOF variants and 58 individuals with GOF variants.

The median age of onset for the M1 proline cohort was 3.5 mo [interquartile range (IQR): 1.0-4.0], which is similar to a median age of onset at 3.0 mo (IQR: 0.37-6.0, Mann–Whitney *U* test; U = 255, *P* = 0.87) for the combined GOF group but different from a median age of onset at 9.5 mo (IQR: 7.0-15; U = 114, *P* < 0.0001) for the combined LOF group (Fig. 2*B*). Likewise, there was no difference in seizure risk profiles between the M1 proline cohort and the combined GOF group (Mantel Cox test, χ^2^ = 1.9; *P* = 0.17) while a clear difference to the LOF group was noted (χ^2^ = 25; *P* < 0.0001). Comparisons of available prominent clinical features revealed a remarkable similarity between the M1 proline cohort and the combined GOF group for severe ID (100% vs. 68%, odds ratio (OR) = 4.6 [95% CI: 0.64-53]; *P* = 0.16), severe movement disorders (40% vs. 37%, OR = 0.89 [0.25-3.1]; *P* = 1.0), hypotonia (80% vs. 65%, OR = 1.2 [0.40-11]; *P* = 0.47), microcephaly (50% vs. 48%, OR = 1.1 [0.24-5.2]; *P* = 1.0), and absence of fever sensitivity [0% vs. 7.8%, OR = Not Determined (ND); *P* = 1.0]. In contrast, observations for the M1 proline cohort differ substantially from the LOF group for the same features: severe ID (100% vs. 15%, OR = 55 [7.2-610]; *P* < 0.0001), severe movement disorders (40% vs. 2.8%, OR = 23 [4.1-130]; *P* = 0.0017), hypotonia (80% vs. 16%, OR = 22 [3.8-110]; *P* < 0.0001), microcephaly (50% vs. 0%, OR = ND; *P* = 0.00060), and fever sensitivity (0% vs. 54%, OR = ND; *P* = 0.00060).

Hence, based on a range of clinical parameters, the clinical outcomes for individuals in the M1 proline cohort align closely with those of individuals with established GABA_A_R GOF variants, but do not resemble those of individuals with established LOF variants.

### Functional Analysis of M1 Proline Mutations.

All 11 individuals in the M1 proline cohort were heterozygous for their genetic variants. Thus, the *GABRA1*, *GABRB2,* and *GABRB3* variants in this cohort can lead to different receptor assemblies containing either zero, one, or two variant α1, β2, or β3 subunits. Under the assumption of similar transcription/translation efficiency of the two alleles, individuals will express 25% receptors with two wildtype subunits, 25% receptors with two variant subunits, and 50% receptors consisting of two distinct stoichiometries containing a wildtype and variant subunit (*SI Appendix*, Fig. S2). By contrast, since the α1β2/3γ2 receptor complex only contains a single γ2 subunit, individuals carrying *GABRG2* variants will express an even mix of wildtype and variant receptors.

To enable interrogation of the functional implications of M1 proline variants in all five subunit positions in the α1β2/3γ2 receptor, the subunit concatenation methodology was applied (29, 30). Essentially, this entails linking all five subunits of a single receptor complex into a single cDNA in the correct order. The order of subunits used in the cDNAs was γ2-β2/3-α1-β2/3-α1 with the γ2 subunit in the 1st construct position and an α1 subunit in the 5th construct position. Upon heterologous expression, this leads to the expression of a fusion protein that can only assemble in the predetermined counterclockwise order (29, 30). Through these constructs, the M1 proline point mutations could be introduced in any of the five subunits as desired, thus enabling the expression of uniform populations of single- or double-mutant receptors. Functional evaluation was performed using two-electrode voltage clamp electrophysiology and three functional parameters were evaluated: GABA sensitivity (EC_50_), maximal GABA-evoked current amplitude (I_max_), and receptor desensitization properties. For receptor desensitization properties both current decay time constants and steady-state current amplitudes were assessed. Detailed data for the electrophysiological experiments are presented in Datasets S2–S4.

### α1 Subunit M1 Proline Mutations.

**Fig. 3.**
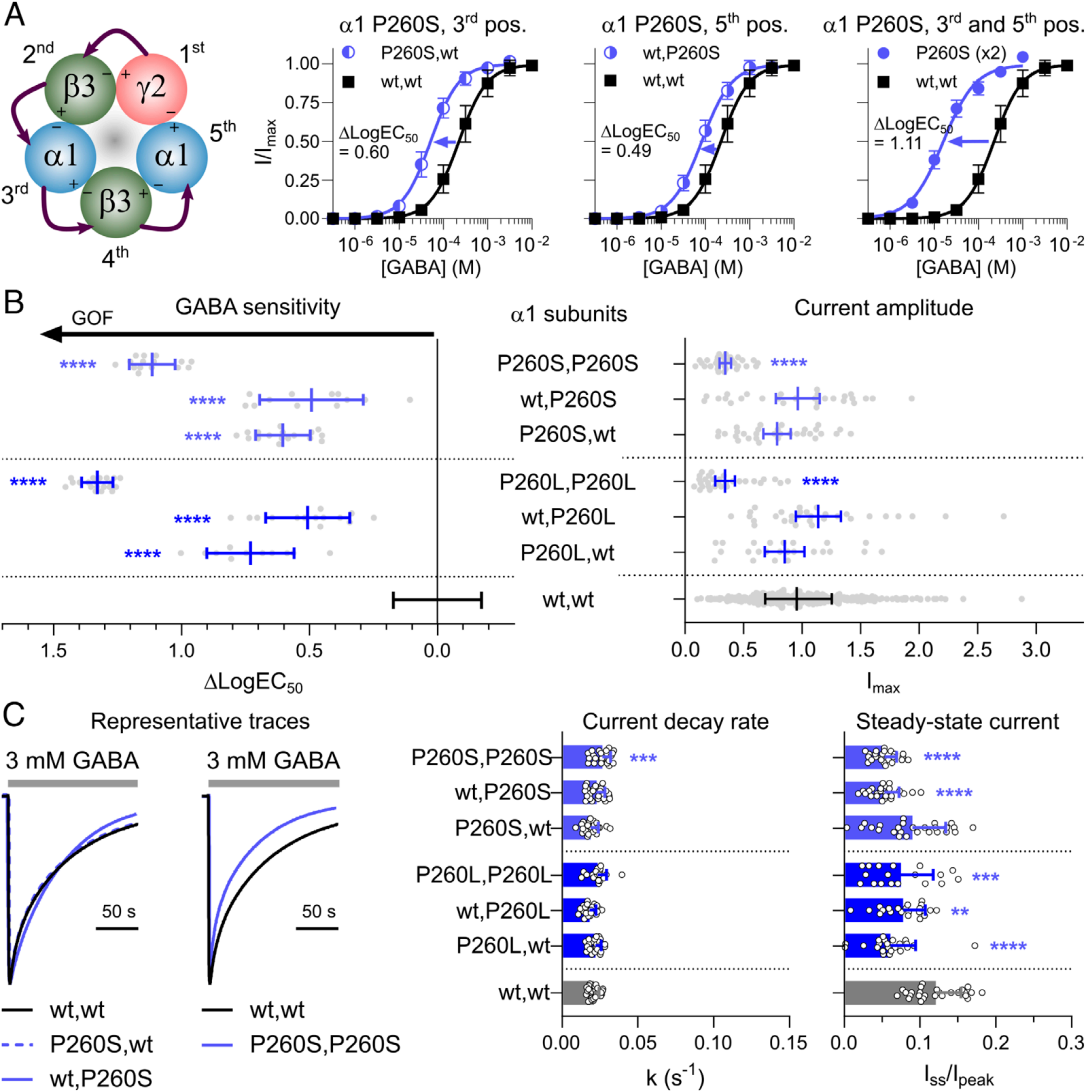
Functional properties of GABA_A_Rs containing M1 proline mutations in the α1 subunit. Variant receptors are compared to the wildtype (wt) receptor. Statistical analyses results are presented as **P < 0.01; ***P < 0.001; ****P < 0.0001. (*A*) α1 subunit mutations are introduced in the 3rd and 5th construct positions of the concatenated receptor. *Middle* and *Right*: GABA concentration–response relationships are presented as mean ± SD for α1β3γ2 GABA_A_Rs containing either one or two α1 P260S mutation(s) with mean differences in LogEC_50_ (ΔLogEC_50_) between variant (n = 11-20) and wt (n = 28) receptors. (*B*) Changes in GABA sensitivity [n = 11-20 (variants) and n = 156 (wt)] and maximal GABA-evoked current amplitudes [n = 24-31 (variants) and n = 332 (wt)] are depicted for receptors containing one or two α1 P260S or P260L mutations. For detailed information, see Datasets S2–S4. (*C*) Desensitization properties are presented as representative traces, current decay rates, and steady-state currents for variant and wt receptors (n = 21-26).

Data for the α1^P260L^ and α1^P260S^ mutations were virtually identical, so only results for the α1^P260S^ mutation are presented in the following. α1β3γ2 receptors with a single α1^P260S^ mutation in the 3rd (P260S, wt) or 5th (wt, P260S) construct position displayed similar increases in GABA sensitivity, with ΔLogEC_50_ values of 0.60 and 0.49, respectively (Fig. 3 *A* and *B*). The receptor with α1^P260S^ mutations in both the 3rd and 5th positions (P260S, P260S) exhibited a much larger increase in GABA sensitivity, with a ΔLogEC_50_ value of 1.11. These changes correspond to 3- to 13-fold increases in GABA sensitivity. Maximal current amplitudes for the two single-mutant receptors were comparable to wildtype receptor amplitudes (Fig. 3*B*). In contrast, the double-mutant receptor displayed reduced I_max_ current amplitudes, constituting approximately 34% of wildtype receptor amplitudes (Fig. 3*B*). This current loss appeared to be partly due to decreased total and cell surface expression levels, but not to changes in GABA gating efficiency (*SI Appendix*, Figs. S3 and S4). Current decay rates were largely unchanged for all three receptor combinations containing the α1^P260S^ mutation, with rate constants of 0.019 to 0.027 s^−1^, compared to 0.021 s^−1^ for the wildtype (Fig. 3*C*). Yet, steady-state currents were reduced for all α1^P260S^-containing receptors, with I_ss_/I_pk_ values of 9.0% (P260S, wt), 5.3% (wt, P260S), and 5.3% (P260S, P260S) compared to the wildtype value of 12%.

Overall, α1 subunit M1 proline mutations (α1^P260L^ and α1^P260S^) primarily led to increased receptor sensitivity to GABA. This effect was observed across all single-mutant receptors, but the difference was even more pronounced in the two double-mutant receptors, indicating an additive impact of multiple mutant α1 subunits. Reductions in maximal current amplitudes were noted only for the double-mutant receptors. Additionally, most variant receptors exhibited reduced steady-state currents during prolonged GABA applications, without affecting the current decay rates.

### β2 and β3 Subunit M1 Proline Mutations.

**Fig. 4.**
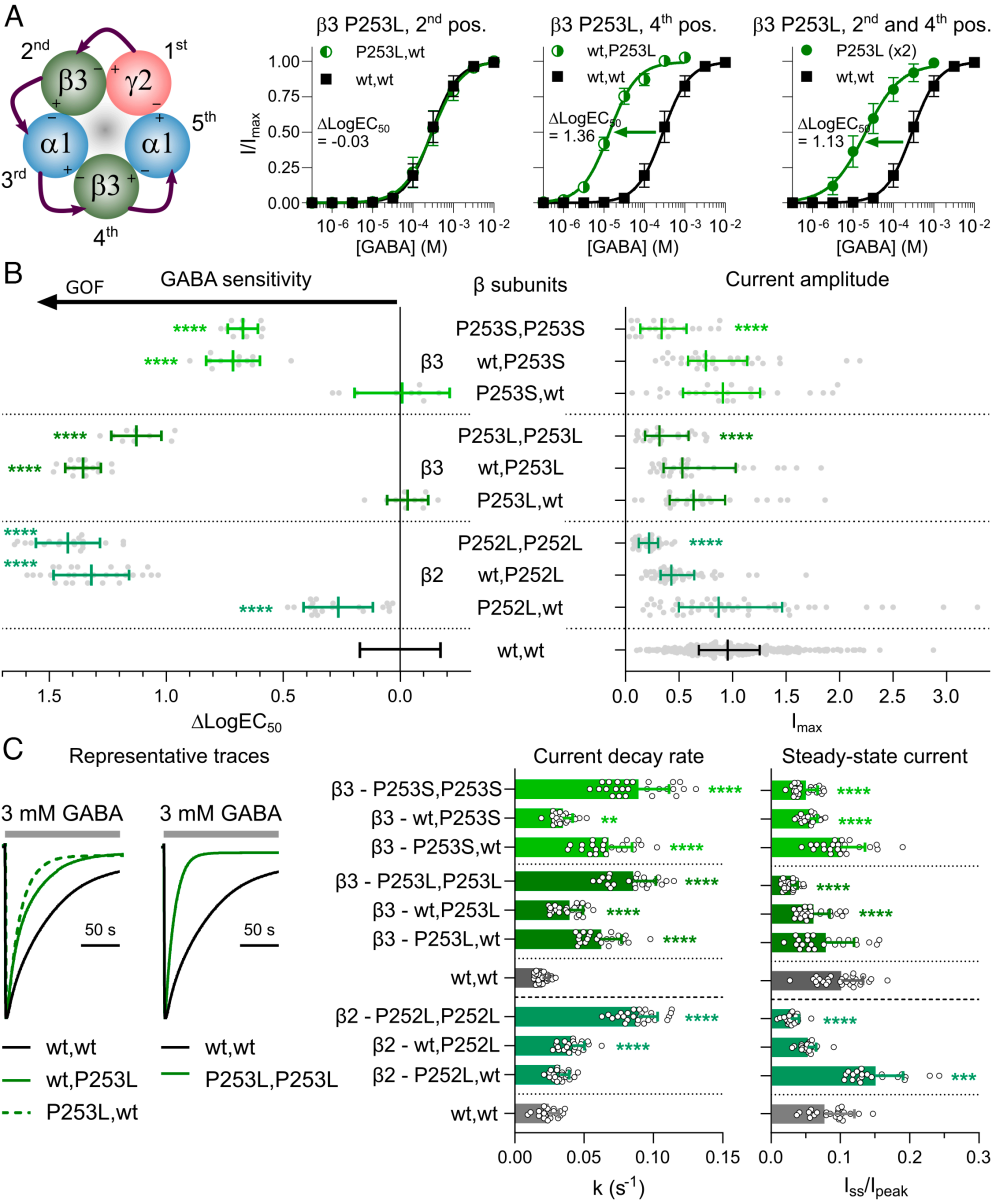
Functional properties of GABA_A_Rs containing M1 proline mutations in the β2 or β3 subunits. See Fig. 3 legend for a description of figure layout and statistical methods. (*A*) β3 subunit mutations are introduced in the 2nd and 4th construct positions of the concatenated receptor. GABA concentration–response relationships are presented for α1β3γ2 GABA_A_Rs containing either one or two β3 P253L mutation(s) with indicated mean difference in LogEC_50_ (ΔLogEC_50_) between the variant (n = 9-15) and wt (n = 29) receptors. (*B*) Changes in GABA sensitivity [n = 9-28 (variants) and n = 156 (wt)] and maximum GABA-evoked current amplitude [n = 22-48 (variants) n = 332 (wt)] are depicted for receptors containing either one or two β2 P252L, β3 P253L, or P253S mutations. For detailed information, see Datasets S2–S4. (*C*) Desensitization properties are presented as representative traces, current decay rates, and steady-state currents for variant and wt receptors (n = 22-28).

α1β3γ2 receptors with a β3^P253L^ mutation in the 2nd (P253L, wt) construct position displayed GABA sensitivity similar to the wildtype (Fig. 4*A*). In contrast, receptors with a β3^P253L^ mutation in the 4th (wt, P253L) position exhibited a remarkable 23-fold increase in GABA sensitivity on par with the receptor carrying two β3^P253L^ mutations (P253L, P253L). Interestingly, the impact of two β3^P253L^ mutations, as indicated by a ΔLogEC_50_ value of 1.13, mirrors the effects seen with two α1 subunit M1 proline mutations (Fig. 3 *A* and *B*). The maximum current amplitude patterns observed for β3^P253L^ mutant receptors were likewise similar to those seen for the α1 mutations. While none of the single-mutant receptors displayed altered maximal current amplitudes, the double-mutant receptor displayed reduced amplitudes with an I_max_ value of 38% of the wildtype level (Fig. 4*B*). With respect to desensitization properties, both single-mutant receptors displayed accelerated current decay kinetics compared to the wildtype receptor, which had a current decay rate of k = 0.020 s^−1^. Notably, the acceleration was more pronounced for the receptor with the mutation in the 2nd construct position with a rate constant of k = 0.062 s^−1^ compared to the 4th position, where the rate constant was k = 0.039 s^−1^ (Fig. 4*C*). As expected, the double-mutant receptor displayed an even higher current decay rate constant of k = 0.085 s^−1^. Steady-state currents remained largely unaffected by the β3^P253L^ mutation in the 2nd construct position, with I_ss_/I_pk_ of 7.9% compared to 10% for the wildtype, while introducing the β3^P253L^ mutation into the 4th position led to a modest reduction in I_ss_/I_pk_, resulting in 6.1%. Once again, the double-mutant receptor exhibited the strongest effects with nearly complete current decay, resulting in a 3.6-fold reduced I_ss_/I_pk_ ratio of 2.8%.

Data obtained for α1β2γ2 and α1β3γ2 receptors containing the β2^P252L^ and β3^P253S^ mutations, respectively, closely resembled those of the β3^P253L^-containing receptors. The most noticeable distinction was a modest increase in GABA sensitivity (ΔLogEC_50_ = 0.26) observed in the receptor with a single β2^P252L^ mutation in the 2^nd^ (P252L, wt) position (Fig. 4*B*).

In summary, introducing two β subunits with M1 proline mutations enhanced the GABA sensitivity of the α1β2/3γ2 receptor. However, this enhancement was accompanied with reduced current amplitudes and accelerated desensitization rates, leading to near-complete desensitization at steady state. Intriguingly, the effects were not symmetrical in single-mutant receptors. Specifically, a single mutation in the 4th position β subunit, flanked by two α1 subunits, increased GABA sensitivity to a degree comparable to the enhancement seen in the double-mutant receptor. In contrast, the other single mutant receptor, flanked by a γ2 and a α1 subunit, showed either no effects or limited functional changes. None of the single β mutant receptors exhibited alterations in current amplitudes, and most single-mutant receptors displayed increased desensitization properties, albeit to a lesser degree than the double-mutant receptors.

### γ2 Subunit M1 Proline Mutations.

**Fig. 5.**
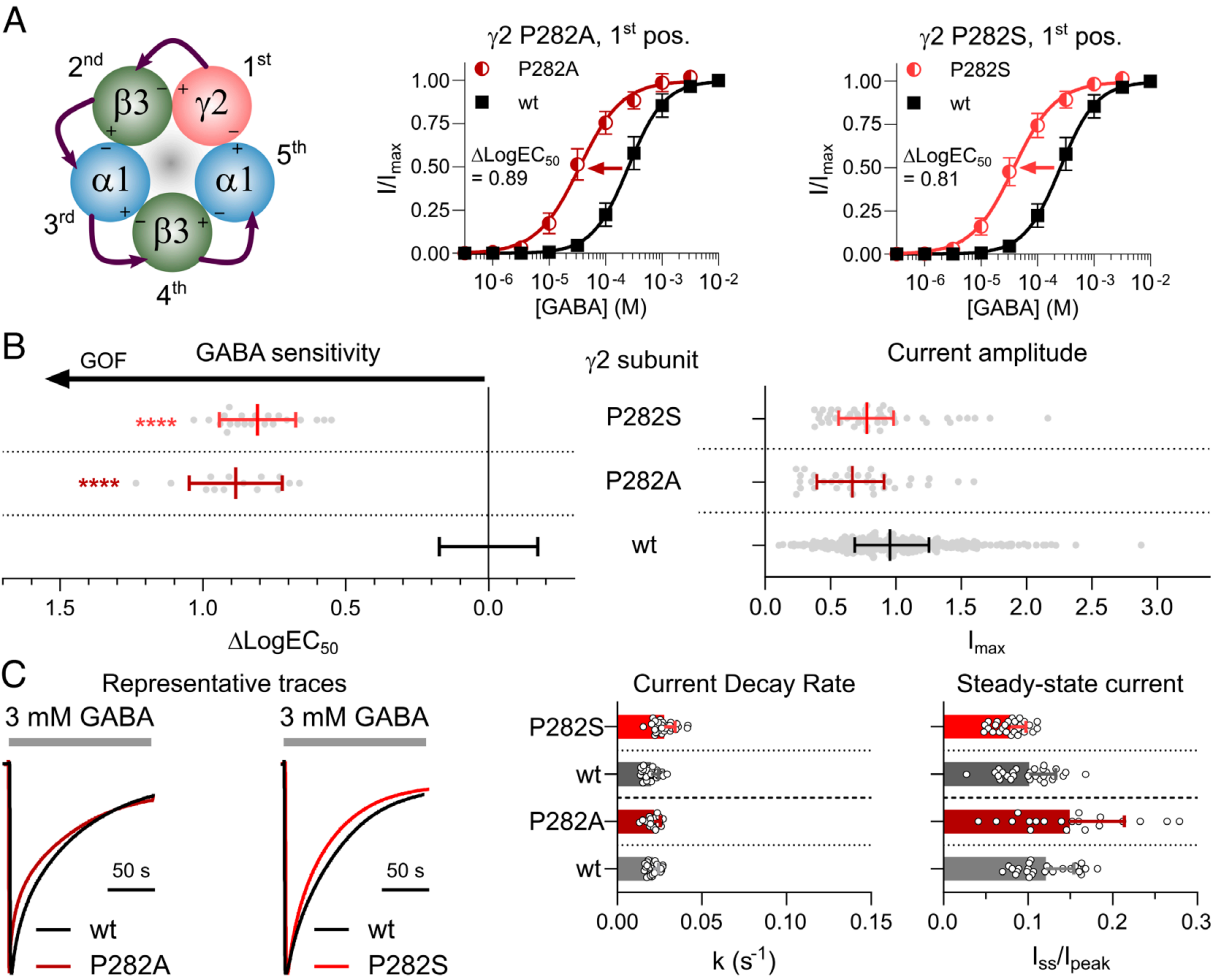
Functional properties of GABA_A_Rs containing M1 proline mutations in the γ2 subunit. See Fig. 3 legend for a detailed description of the figure layout and statistical methods. (*A*) γ2 subunit mutations are introduced in the 1st construct position of the concatenated receptor. GABA concentration–response relationships are displayed for α1β3γ2 GABA_A_Rs containing γ2 P282A (n = 14) or P282S (n = 21) mutations and wildtype receptors (n = 47). (*B*) Changes in GABA sensitivity [n = 14-21 (variants) and n = 156 (wt)] and maximum GABA-evoked current amplitude [n = 36-48 (variants) and n = 332 (wt)] are depicted for receptors containing the γ2 P282A or P282S mutation. For detailed information, see Datasets S2–S4. *(C)* Desensitization properties are presented as representative traces, current decay rates, and steady-state currents for variant and wt receptors (n = 20-27).

α1β3γ2 receptors with γ2^P282A^ and γ2^P282S^ mutations in the 1st construct position displayed similar increases in GABA sensitivity compared to wildtype with ΔLogEC_50_ values of 0.88 and 0.81, respectively (Fig. 5*A*). No changes were observed in the maximal current amplitudes (Fig. 5*B*). Moreover, both the two mutant receptors displayed wildtype-like current decay rates and steady-state currents with k values of 0.022 to 0.028 s^−1^ vs. 0.021 s^−1^ for the wildtype and I_ss_/I_pk_ values of 7.9 to 15% vs. 12% for the wildtype (Fig. 5*C*). Thus, incorporating M1 proline mutations in the γ2 subunit markedly increases the GABA sensitivity but has no impact on maximal current amplitudes and desensitization properties.

### Molecular Dynamics Simulations.

In the experiments above, the functional effects of M1 proline mutations in the three subunit types appeared to be independent of whether the proline was mutated to alanine, serine, or leucine. To investigate this further, we conducted molecular dynamics simulations to examine the effects of M1 proline variants on the GABA-bound α1β2γ2 GABA_A_R structure (PDB: 6X3Z). Five simulations were performed in triplicate to individually study the impact of the following mutants: α1^P260L^, α1^P260S^, β2^P252L^, and γ2^P282A^ in addition to the wildtype.

First, the stability of the protein was assessed using RMSD, computed from Cα positions relative to the initial conformation. The RMSD plots indicated that the protein exhibited the largest conformational changes during the first 200 ns and then stabilized (*SI Appendix*, Fig. S5). Both the wildtype and variant-bearing proteins showed similar average RMSD values (around 3.2 Å) after the initial 200 ns. The initial conformational change corresponded with the channel pore becoming more closed relative to the published structure (*SI Appendix*, Fig. S6). A secondary structure analysis of the M1 helix revealed that residues in the helical turn directly above the M1 proline in α1 and γ2 subunits (α1 F253-T257 and γ2 F275-T279) exhibited mostly π-helical character (*SI Appendix*, Fig. S7). These secondary structure propensities were similar, regardless of the subunit investigated or the presence/absence of a variant. The helix region in the β2 subunit (F245-T249) also displayed π-helical character. However, in the 2nd subunit position, the β2^P252L^-containing structure displayed more loop-like character (greater disorder) while the structure became mostly π-helical in the 4th subunit position.

**Fig. 6.**
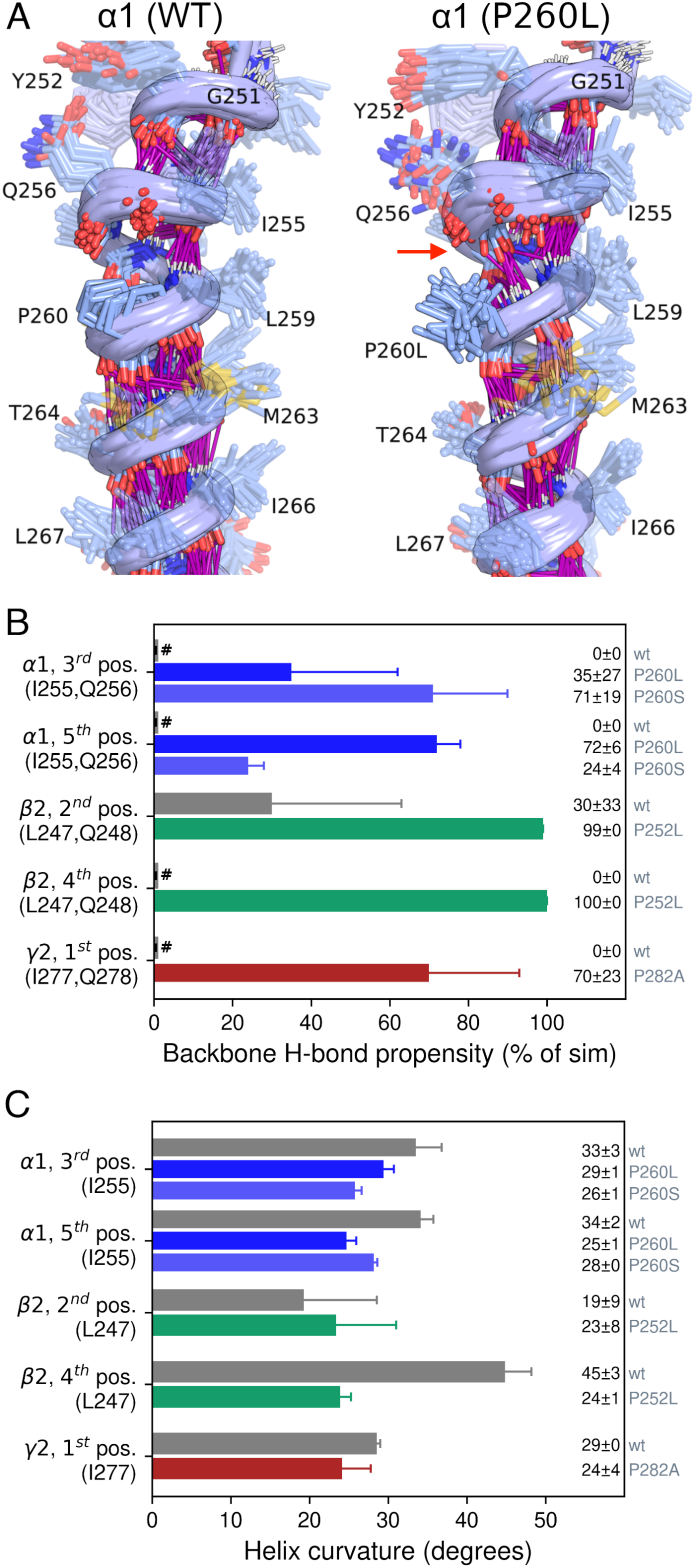
Analysis of the M1 helix curvature from 500 ns MD simulations of the α1β2γ2 GABA_A_R. (*A*) An ensemble of simulation frames for the α1 subunit M1 helix in the 3rd position with wildtype sequence (*Left*) and with the P260L mutant (*Right*), 10 frames are captured uniformly over the last 300 ns of three simulation replicas (30 frames in total). Backbone H-bonds are shown as purple lines, protein sidechain atoms except for P260 (*Left*) and P260L (*Right*) are faded for clarity. The red arrow highlights the ability of Q256 to form backbone H-bond in the M1 helix containing P260L but not wildtype M1 helix. (*B*) H-bond propensity was measured as percentage of simulation frames where H-bonds occurred from the backbone carbonyl oxygen of α1 I255/Q256, β2 L247/Q248, and γ2 I277/Q278 to NH atoms below them. H-bonds were measured as an interaction to either but not both residues, # denotes near-zero value (no H-bonds observed). (*C*) Helix curvature around α1 I255, β2 L247, and γ2 I277. For both plots, means ± SD are obtained from the last 300 ns of triplicate MD simulations.

Visual analysis of the simulations revealed that the π-helix section of M1 corresponded with a helical bulge (Fig. 6*A*). H-bond analysis demonstrated that the M1 proline prevented upstream π-helical residues (α1 I255/Q256, β2 L247/Q248, and γ2 I277/Q278) from forming H-bonds through their backbone carbonyl oxygen atoms (Fig. 6*B* and *SI Appendix*, Figs. S8–S11). Only the β2 subunit in the 2nd subunit position was able to form backbone H-bonds to some degree. Notably, all variants in all subunit positions increased H-bond propensity compared to the wildtype. The effects were pronounced for the mutant α1^P260L^ and α1^P260S^ as well as γ2^P282A^ subunits with H-bond propensities ranging from 24 to 72%, compared to no H-bonds observed in wildtype receptors. Perhaps the most striking effect was noted for the mutant β2^P252L^ subunit in the 4th position, with a 100% H-bond propensity compared to no H-bonds for the wildtype. The mutated β2^P252L^ subunit in the 2nd position showed a threefold increase in H-bond propensity from 30% for the wildtype to 99% for the mutant. Additionally, the ability to form these H-bonds made the M1 helix less curved (Fig. 6 *A* and *C*). Helix curvature peaked around the same position in all subunits (α1 I255, β2 L247, and γ2 I277; *SI Appendix*, Fig. S12). For the α1 subunit, the 3rd position M1 helical curvature was reduced in α1^P260L^ (29°) and P260S (26°) compared to the wildtype (33°), respectively. Similar trends were seen for the remaining variants except for the mutant β2^P252L^ subunit in the 2nd position.

Hence, the molecular dynamics simulations revealed that functional effects of M1 proline mutations across subunit types were independent of whether the proline was mutated to alanine, serine, or leucine. Additionally, all variants increased local H-bond propensity compared to the wildtype, affecting helix curvature and stability.

## Discussion

Unlike the wide clinical heterogeneity commonly observed in individuals with GABA_A_R variants, all 11 individuals with paralogous M1 proline variants exhibited a remarkably similar clinical phenotype irrespective of which subunit harbored the variant (Fig. 2). Such consistent clinical presentation would generally be expected to result from similar functional changes caused by the various variants. When comparing the M1 proline cohort to established *GABRA1, GABRB2*, and *GABRB3* cohorts, where both GOF and LOF variants have been identified (3–5), the clinical observations in the M1 proline variant cohort align closely with observations for GOF variants. Hence, it would be expected that M1 proline variants also cause GOF. While this was confirmed, the underlying functional data revealed surprising complexities.

### Functional Consequences of a M1 Proline Mutation in a Single Subunit.

Receptors with γ2^P282A^ and γ2^P282S^ mutations represented the simplest case and displayed increased GABA sensitivity with no change to maximal current amplitudes or desensitization properties (Fig. 5). Similar observations were seen for single-mutant receptors with α1^P260L^ or α1^P260S^ mutations (Fig. 3). Hence, the molecular phenotype of receptors with single-subunit M1 proline mutations in the γ2 or α1 subunits can be classified as GOF without ambiguity. Extrapolating this into expected receptor populations in heterozygous individuals, 50% of expressed receptors would be expected to have GOF properties in *GABRG2* individuals and at least 50% of expressed receptors to have GOF properties in *GABRA1* individuals since both single-mutant receptor combinations are equally affected.

For receptors with β2^P252L^, β3^P253L^, and β3^P253S^ mutations, however, there was an intriguing asymmetric contribution to increases in GABA sensitivity (Fig. 4). Furthermore, desensitization properties for most receptors with M1 proline mutations in β subunits were characterized by increased current decay and/or reduced steady-state currents, which is typically considered a LOF trait. Single-mutant receptors with β2/3 mutations in the 4th position thus display mixed GOF and LOF characteristics, whereas receptors with β2/3 mutations in the 2nd position display an almost wildtype-like molecular phenotype. Consequently, only half of the expressed single-subunit mutated receptors in *GABRB2/B3* individuals would be expected to display a clear GOF property.

### Functional Consequences of M1 Proline Mutations in Two Subunits.

Receptors with two α1^P260L^ or α1^P260S^ mutations displayed profound increases in GABA sensitivity (Fig. 3). This increase approximately represented the sum of the changes observed for single-mutant receptors, suggesting an additive effect of introducing additional mutated subunits. Receptors with two β2^P252L^, β3^P253L^, or β3^P253S^ mutations likewise displayed profound increases in GABA sensitivity, which were primarily driven by effects of the 4th position β2/3 mutation (Fig. 4). All double-mutant receptors displayed reduced GABA-evoked maximal current amplitudes, which given an unaltered gating efficiency appear to be partly rooted in reduced surface expression levels (*SI Appendix*, Figs. S3 and S4). Furthermore, desensitization properties for receptors with β mutations were characterized by both increased current decay and reduced steady-state currents. Overall, these findings agree with previous observations. Greenfield, Zaman (31) found that alanine substitutions of the GABA_A_R α1 or β1 subunit M1 prolines increased GABA sensitivity by 3- to 10-fold, while also reducing the maximum GABA-evoked current amplitudes in double-mutant α1β1γ2 receptors.

By applying the common assumption that partial loss of maximal current amplitude reflects a LOF molecular phenotype, double-mutant receptors with α1 or β2/3 subunit M1 proline mutations thus display mixed GOF and LOF characteristics. While up to 25% double-variant receptors would be expected in heterozygous carriers of *GABRA1*, *GABRB2,* or *GABRB3* variants, the observation of current loss makes it difficult to gauge the degree by which such receptors are present in synapses.

### Increased GABA Sensitivity as the Key Driver of M1 Proline Variant Pathogenicity.

The similar clinical outcomes observed in individuals with paralogous M1 proline variants allow us to infer the most critical functional parameter for the clinical phenotypes. All individuals exhibit the hallmarks of GOF variants, which coincides with the observation that increased GABA sensitivity is the only consistent functional trait across all paralogous variants. This suggests that GABA sensitivity plays a pivotal role in determining the clinical phenotype within this cohort. These findings align with previous studies involving GABA_A_R variants that demonstrated clear correlations between clinical phenotypes and changes in GABA sensitivity (3–5). Some receptors additionally displayed reduced maximal current amplitudes or increased desensitization characteristics, which have previously been associated with LOF variants (12, 26, 32–34). Nevertheless, our data reveal that neither a reduction in maximal current amplitudes nor an increase in desensitization properties should be extrapolated to indicate an overall LOF effect in cases where the GABA sensitivity is also substantially increased.

Reduced current amplitudes observed in double-mutant receptors have previously led to the classification of *GABRA1* and *GABRB2* M1 proline variants as LOF variants (9, 10). However, as shown in this study, reduced current amplitude in double-mutant receptors does not necessarily imply the same effect in single-mutant receptors, which would be expected to constitute a much larger proportion of the total receptor pool. This underscores the importance of considering the contribution of single-mutant receptors in functional assessments of GABA_A_R variants, and literature studies that do not take these issues into account may not present reliable sources of functional data.

### Using Paralogous Variants as Predictors in GABA_A_Rs.

While the observations for the M1 proline variants suggest that paralogous variant information can be used to predict functional phenotypes, this approach should not be indiscriminately applied to all scenarios. The pseudosymmetrical structure of GABA_A_Rs and the unique roles of different subunit types in receptor functionality must also be considered. In regions like the channel pore, highly conserved residues are likely to contribute uniformly to activation across subunit types. However, this uniformity does not extend to all parts of the extracellular domain, with the GABA-binding pocket being the most prominent example. Finally, in other regions with low sequence identity and limited structural information, such as the intracellular domain, it is meaningless to discuss paralogous variants. Therefore, we suggest that information from paralogous variants may be applied for conserved residues within the channel pore helices for all subunit types and within individual subunit classes in the extracellular domain but is not advisable for use in the intracellular domain (*SI Appendix*, Fig. S13). Additionally, it is important to note that this application should only be used in situations where the functional data are from a well-established assay and where there are similarities between the clinical indicators.

### Synaptic vs. Extrasynaptic Receptors.

Previously, we described a paralogous M1 proline variant in the *GABRD* gene, and similarly to the variants in this study, the δ subunit P257L variant caused a GOF molecular phenotype (8). In δ-containing receptors, the δ subunit substitutes for the γ2 subunit, which has a profound impact on intrinsic receptor characteristics as well as on neuronal distribution. While γ2-containing receptors primarily are localized to postsynaptic densities, δ-containing receptors are found in extrasynaptic spaces where they respond to low ambient concentrations of GABA and spillover from synaptic activity (35). Furthermore, δ-containing receptors often associate with the α4 subunit, and recently a M1 proline P266L variant was described in *GABRA4* (36). Although no functional data were presented, based on our results for paralogous variants, it is reasonable to assume that this variant also causes GOF. Interestingly, individuals harboring the *GABRD* P257L and *GABRA4* P266L variants exhibited milder clinical phenotypes (e.g., mild ID, seizure free, and ambulant) compared to those with the M1 proline variants in this study. This might suggest that the clinical outcome of a GOF molecular phenotype is less severe for extrasynaptic δ-containing than for synaptic γ2-containing receptors. However, these observations are based on only two individuals, and other GOF variants in *GABRD* and *GABRA4* have been associated with more severe clinical phenotypes (7, 8).

### Asymmetric Effects of the M1 Proline Residue on GABA_A_R Function.

A proline residue is a unique amino acid due to the incorporation of its α-nitrogen into a pyrrolidine ring. This introduces structural restraints on the peptide backbone and eliminates the ability for the nitrogen to act as a hydrogen donor. Consequently, proline residues can cause a “kink” to the backbone in α-helical structures, which is precisely what is observed for the M1 proline (Fig. 6). Molecular dynamics simulations reveal that this kink can be alleviated by the assessed proline mutations. In support of the functional data, the outcome is remarkably consistent across various variants and subunits, emphasizing that the effects primarily result from the absence of the proline residue, rather than the specific amino acid introduced. Notably, the simulations also revealed positional differences. For instance, the effects of the β2 subunit in the 2nd position are more chaotic. Backbone hydrogen bonding in the vicinity of the M1 proline in the wildtype receptor is observed only for the β2 subunit in the 2nd position, and this subunit does not exhibit changes in helix curvature upon mutation. These asymmetrical effects of M1 proline mutations likely arise from different local helical structures at various positions. Given that proline mutations significantly impact desensitization compared to other subunits, it is tempting to speculate that β subunit M1 prolines play a special role in promoting allosteric transitions favoring the open and/or desensitized states, consistent with the role this residue plays in related ligand-gated ion channels (37).

Prior studies have also highlighted asymmetric effects of mutations in GABA_A_Rs. For example, ion selectivity appears to be controlled primarily by β subunits, but not by α1 and γ2 subunits (38). Additionally, a recent study on concatenated α1β2γ2 receptors revealed that mutations in the M3 region led to asymmetric effects (39). In that study, γ2 mutations were particularly sensitive to accelerated current decay, which appears to diverge from the findings in this study. Hence, caution should be exercised not to draw overly general conclusions regarding current decay based solely on analyses of a single mutation.

### Limitations.

This study has several limitations. First, the M1 proline cohort included a small number of individuals, which could affect the generalizability of our findings. Additionally, clinical information relied on retrospective data from published sources, medical records, and interviews, introducing potential heterogeneity in data collection and analysis. Furthermore, in comparing the M1 proline cohort with established cohorts, a search criterion was set that required the established cohorts to include both individuals with GOF and LOF variants, as well as a detailed phenotypic description. This criterion might have excluded smaller studies. Last, it is important to acknowledge that in vitro expression systems, while valuable, do not fully replicate the intricate neuronal processes occurring in vivo. Therefore, the assay employed here may not account for all relevant factors influencing receptor function.

## Conclusion

As the field is progressing toward personalized medicine approaches that include both better symptomatic and disease-modifying treatments, it is imperative to know the functional outcomes of variants. Here, we demonstrate that paralogous variants affecting the 100% conserved proline residue in the M1 transmembrane helix of GABA_A_R subunits lead to GOF traits. Notably, this conclusion is based on both the clinical phenotypes and functional analyses. Functional analyses also revealed complex, additive, and sometimes asymmetric effects of the variants on GABA_A_R function. Consequently, a minority of receptors in an individual may exhibit divergent effects compared to most expressed receptors complicating the determination of the molecular phenotype. This issue underscores the importance of analyzing the most common receptors and likely applies to all receptor and channel types assembled from multiple subunits such as glutamate receptors and potassium channels.

## Materials and Methods

### Genetic Landscape.

Individuals with M1 proline variants in *GABRA1, GABRB2, GABRB3,* or *GABRG2* were included for clinical and functional assessment. The total cohort included 11 individuals; 2 previously unpublished, 2 previously published for whom additional clinical information was obtained (26, 28), and 7 from the literature (9–11, 24, 25, 27). Clinical information from the two previously unpublished cases was collected directly from the parents of the affected children. The clinical data were evaluated by a team of skilled epileptologists and epilepsy geneticists, and epilepsy syndromes were classified according to the most recent ILAE classification (19). Data are reported in line with the Strengthening Reporting of Observational Studies in Epidemiology (STROBE) statement.

### Protocol Approvals, Registrations, and Consent.

The study was conducted according to the ethical principles for medical research outlined in the Declaration of Helsinki. The study was approved by the Institutional Review Board at the Danish Epilepsy Centre, Filadelfia (EMN-2024-01998), and were performed with informed consent of individuals or their responsible relatives.

### Functional Analysis.

Extended method descriptions are presented in *SI Appendix*, Fig. S14. Briefly, concatenated wildtype γ2-β2-α1-β2-α1 and γ2-β3-α1-β3-α1 constructs were created as previously described (29, 30). From these, mutant subunit constructs were created using standard restriction digest and ligation protocols (40). Final constructs were subjected to diagnostic restriction digestion and Sanger sequencing. *Xenopus laevis* oocytes were microinjected with ~25 ng of cRNA and incubated at 18 °C for at least 48 h. Receptor function was measured using two-electrode voltage clamp electrophysiology (4, 5, 34). GABA sensitivity (EC_50_), maximal GABA-evoked current amplitudes (I_max_), and desensitization characteristics [current decay rate (k) and steady-state currents (I_ss_/I_pk_)] were assessed. Assays followed Brnich et al.’s (41) recommendations, with experiments done in parallel in at least two oocyte batches, including wildtype controls.

### Molecular Dynamics Simulations.

Extended method descriptions are presented in *SI Appendix*, Fig. S14. The CHARMM-GUI webserver was used to create solvated membrane systems for the wildtype GABA-bound α1β2γ2 GABA_A_R structure (PDB: 6X3Z) and separate systems for the protein with the M1 proline mutants: α1^P260L^, α1^P260S^, β2^P252L^, and γ2^P282A^ (42, 43). Each system was converted to united-atom representation (44, 45) and simulated using GROMACS 2021.4 (46) for 500 ns in triplicate following equilibration. For the protein, Cα atom RMSD and the pore radius profile were analyzed, for the M1 helix, backbone H-bond propensity, secondary structure propensity, and helix curvature were analyzed (47–50).

### Estimated Maximal Open Probability and Receptor Cell Surface Expression.

Extended method descriptions are presented in *SI Appendix*, Fig. S14. Estimated maximum open probability of wildtype and mutant receptors was obtained using pharmacological modulation with etomidate as previously described (30, 34, 40, 51, 52). Total and cell surface expression were obtained using an enzyme-linked immunosorbent assay (53, 54).

### Statistical Analysis.

All statistical analyses were performed using GraphPad Prism 10.0.1. ΔLogEC_50_ values were compared using one-way ANOVA with Dunnett’s post hoc test with a *P* < 0.0001 threshold. I_max_ current values were compared using the Mann–Whitney *U* test with a *P* < 0.0001 threshold. Desensitization k and I_ss_/I_pk_ values were compared using one-way ANOVA (Kruskal–Wallis rank sum test) and Dunn’s post hoc test. Age of seizure onset and seizure risk comparisons were performed using Mann–Whitney *U* and Mantel-Cox tests, respectively. OR analysis for clinical features was performed using two-tailed Fisher’s exact test.

## Supplementary Material

Appendix 01 (PDF)

Dataset S01 (XLSX)

Dataset S02 (XLSX)

Dataset S03 (XLSX)

Dataset S04 (XLSX)

## Data Availability

Phenotypic information for the cohort is available in Dataset S1. Potential further information from our clinical database will be made available to those eligible. Data will be stored for a minimum of 7 y. Raw data used for all functional analysis are available in Datasets S2–S4. All other data are included in the manuscript and/or supporting information.
