# Supplementary material for: Understanding paralogous epilepsy–associated GABAA receptor variants: Clinical implications, mechanisms, and potential pitfalls
Source: Proc Natl Acad Sci U S A. 2024 Dec 6;121(50):e2413011121. doi: 10.1073/pnas.2413011121 (PMC11648851; doi:10.1073/pnas.2413011121)
Supplement: Supplementary file 1 — Appendix 01 (PDF) [file pnas.2413011121.sapp.pdf]

Understanding paralogous epilepsy associated $GABA_A$ receptor variants: clinical implications, mechanisms and potential pitfalls

Supplementary Information SI1-14

Anthony S. H. Kan*, Ali S. Kusay*, Nazanin A. Mohammadi*, Susan X. N. Lin, Vivian W. Y. Liao, Gaetan Lesca, Sabrine Souci, Mathieu Milh, Palle Christophersen, Mary Chebib, Rikke S. Møller, Nathan L. Absalom, Anders A. Jensen and Philip K. Ahring

Contents:

SI1. Clinical highlights of the M1 proline patient cohort. 2
SI2. Expected receptor expression pattern in heterozygous carriers.......... 3
SI3. Expression properties of wildtype and M1 proline mutated α1β2/3γ2 $GABA_A$Rs. 4
SI4. Maximal open probability estimation of wildtype and M1 proline mutated α1β2/3γ2 $GABA_A$Rs. 6
SI5. RMSD of the α1β2γ2 $GABA_A$ receptor Cα atoms. 7
SI6. Pore radius profile of the α1β2γ2 $GABA_A$ receptor. 8
SI7. Secondary structure (SS) propensity for M1 helix residues. 9
SI8. H-bond analysis of the M1 helix backbone of the α1 subunit, 3rd position. 10
SI9. H-bond analysis of the M1 helix backbone of the α1 subunit, 5th position. 11
SI10. H-bond analysis of the M1 helix backbone of the β2 subunit. 12
SI11. H-bond analysis of the M1 helix backbone of the γ2 subunit. 13
SI12. Curvature profile of the M1 helix tip. 14
SI13. $GABA_A$R subunit sequence homology and recommendations when comparing paralogous variants 15
SI14. Materials and Methods extended version 16

SI1. Clinical highlights of the M1 proline patient cohort.

#	Sex/age	Gene / Variant	Subunit	Age of onset	Seizure type	Syndrome	Intractable epilepsy	DD/ ID
1	F/8y	*GABRA1*/ P260L	α1	3.5m	Tonic, Spasms, Myoclonic	EIDEE	Yes	DD/ Severe ID
2	F/5y11m	*GABRA1*/ P260L	α1	1m	Spasms, Atonic seizure	IESS	Yes	DD/ Severe ID
3	F/3y1m	*GABRA1*/ P260S	α1	4m	Spasms	IESS	No	DD/ Severe ID
4	F/17y	*GABRA1*/ P260S	α1	3m	Spasms, FoS, GTCS	IESS	Yes	DD/ Severe ID
5	M/72m	*GABRB2*/ P252L	β2	2m	Myoclonic, SE, Spasms, Tonic, GTCS	EIDEE	Yes, daily sz	Moderate DD/ Severe ID
6	M/22y	*GABRB3*/ P253L (mosaic 20%)	β3	10m	FoS, GTCS, Atonic, Tonic	DEE	Yes, 2-5 sz daily	Normal/ Severe ID
7	M/6y	*GABRB3*/ P253S	β3	4m	FoS, sGTCS, SE	DEE	Yes, clusters of seizures every 2m	DD/ Severe ID
8	M/13y	*GABRB3*/ P253S	β3	1d	FoS, Focal atonic, sGTCS, SE	EIDEE	Yes, daily sz	DD/ Severe ID
9	M/1y6m	*GABRB3*/ P253S	β3	3.5m	Clusters of FoS	DEE	No Seizure free 10m	DD/ NR
10	F/10y	*GABRG2*/ P282S	γ2	12m	sGTCS, Atypical absences	DEE	Yes	DD/ Severe ID
11	M/18y	*GABRG2*/ P282A	γ2	1d	Trembling, Focal, Myoclonic	EIDEE	Yes, clusters of sz every 3m	Severe DD/ Severe ID

Table SI1. Clinical highlights of the M1 proline cohort. DD: Developmental delay, DEE: Developmental and epileptic encephalopathy, EIDEE: Early-infantile epileptic encephalopathy, FOS: Focal seizure, GTCS: Generalised tonic-clonic seizure, ID: Intellectual disability, IESS: Infantile epileptic spasms syndrome, NR: Not relevant, SE: Status epilepticus, sGTCS: Secondarily generalised tonic-clonic seizure

SI2. Expected receptor expression pattern in heterozygous carriers.

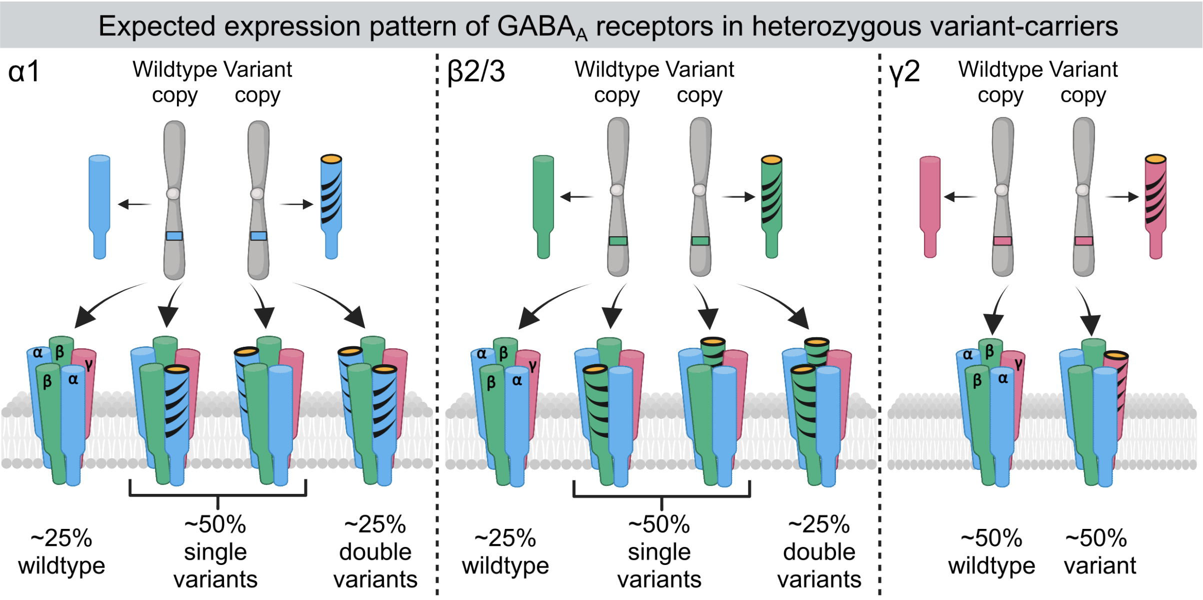

Figure SI2. Expected receptor expression pattern in heterozygous carriers. Schematic diagram showing the proportion of wildtype- and variant-containing receptor expression in heterozygous carriers of *GABRA1*, *GABRB2/B3*, or *GABRG2* variants, assuming a binomial distribution.

SI3. Expression properties of wildtype and M1 proline mutated α1β2/3γ2 GABA$_A$Rs.

To investigate expression levels, α1β2γ2 GABA$_A$Rs containing HA-tagged α1 wildtype or α1^{P260L} subunits were transiently expressed in tsA201 cells, and cell surface and total expression levels were determined by an ELISA. Robust cell surface expression levels were observed when HA-tagged α1 subunits were paired with untagged complementary subunits (Figure SI3A, left). A two-way ANOVA revealed significant differences between receptor type (wildtype or variant) and cDNA quantities. Thus, cell surface expression levels of α1^{P260L}-containing receptors were lower than that of the wildtype receptor. The most pronounced effect occurred at the lowest cDNA quantity of 0.11 µg, where the expression level constituted approximately 45% of the wildtype receptor. Introduction of three additional M1 proline mutations (i.e., in the two β2 subunits and in the γ2 subunit) in the α1β2γ2 receptor did not further reduce cell surface expression levels. Parallel experiments quantifying total expression levels revealed overall similar effects of M1 proline mutations (Figure SI3A, right). Robust cell surface expression levels were likewise observed when HA-tagged α1 wildtype β2 or β2^{P252L} subunits were paired with untagged complementary subunits (Figure SI3B). While the observations with HA-tagged β2 subunits were less marked that those for HA-tagged α1 subunits the trends were the same.

Overall, these data suggest, that M1 proline mutations moderately decrease total and cell surface expression levels, possibly due to lower cellular expression or increased intracellular degradation of the mutated subunits.

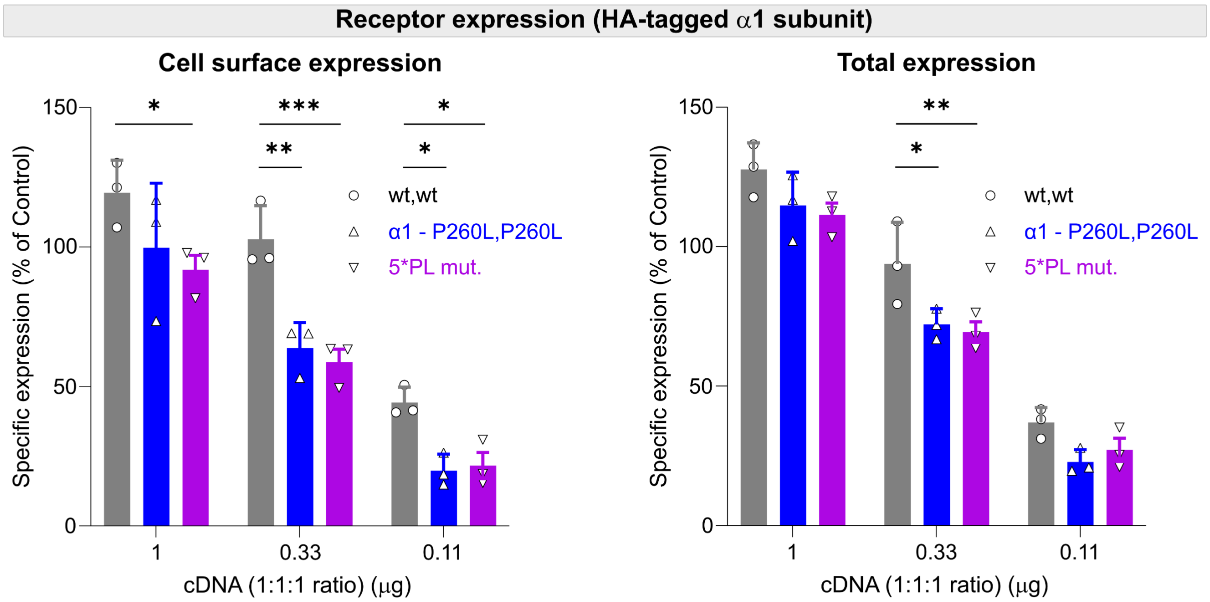

Figure SI3A. Cell surface expression levels of M1 proline mutated α1 subunits in α1β2γ2 GABA$_A$ receptors. To determine cell surface and total expression levels of wildtype α1- and α1 P260L-containing α1β2γ2 receptors, ELISA experiments were performed with tagged α1^{HA} co-expressed with untagged β2 and γ2 subunits in tsA201 cells. Cells were transfected with three different cDNA amounts (1, 0.33 or 0.11 µg in a 1:1:1 subunit ratio, supplemented with empty vector to a total cDNA quantity of 4 µg). Data are based on three independent experiments (each performed in triplicate) and are given

as mean ± SD normalised to control cells transfected with 4 μg cDNA. Left, a two-way ANOVA revealed no significant interaction between α1^{HA}β2γ2 (wt,wt), α1^{HA,PL}β2γ2 (α1 - P260L,P260L) and α1^{HA,PL}β2^{PL}γ2^{PL} (5*PL mutation) receptor combinations ($F_{(4,18)}$ = 0.96; P = 0.45) but significant differences between receptor type and cDNA quantities ($F_{(2,18)}$ = 99 and $F_{(2,18)}$ = 20; both $P < 0.0001$). Right, a two-way ANOVA revealed no significant interaction between receptor combinations ($F_{(4,18)}$ = 0.63; P = 0.65) but significant differences between receptor type and cDNA quantities ($F_{(2,18)}$ = 240 and $F_{(2,18)}$ = 11; $P < 0.0001$ and P = 0.0008, respectively). Indicated significance levels represent Dunnett's multiple comparison test with *: $P < 0.05$, **: $P < 0.01$ and ***: $P < 0.001$.

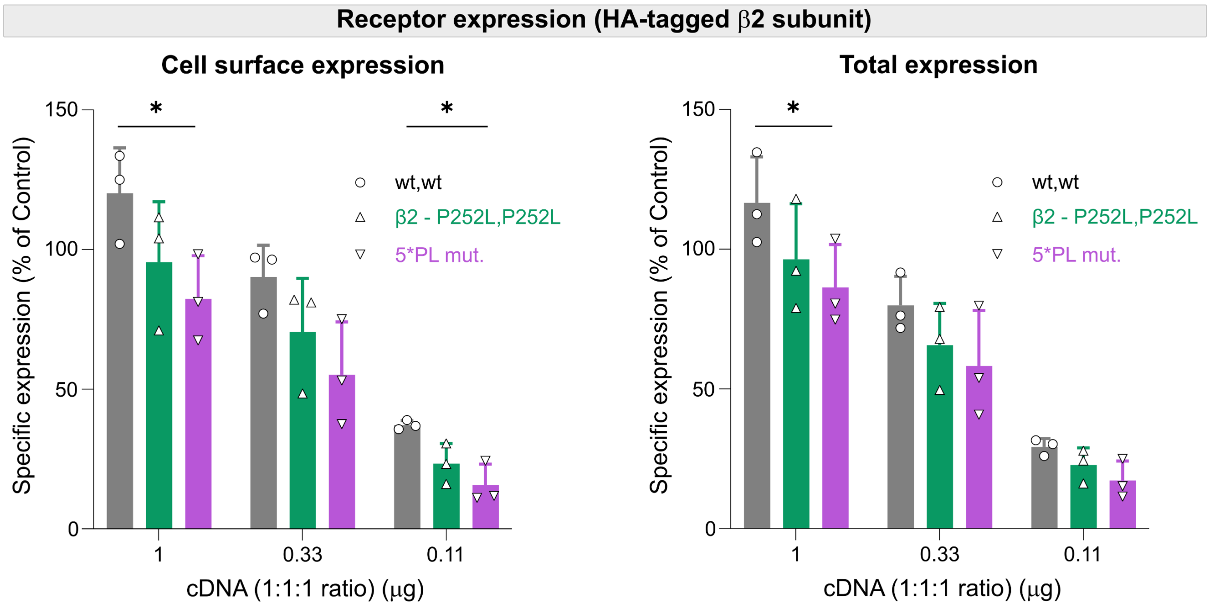

Figure SI3B. Cell surface expression levels of M1 proline mutated β2 subunits in α1β2γ2 GABA$_A$ receptors. To determine cell surface and total expression levels of wildtype β2- and β2 P252L-containing α1β2γ2 receptors, ELISA experiments were performed with tagged β2^{HA} co-expressed with untagged α1 and γ2 subunits in tsA201 cells. Cells were transfected with three different cDNA amounts (1, 0.33 or 0.11 μg in a 1:1:1 subunit ratio, supplemented with empty vector to a total cDNA quantity of 4 μg). Data are based on three independent experiments (each performed in triplicate) and are given as mean ± SD normalised to control cells transfected with 4 μg cDNA. Left, a two-way ANOVA revealed no significant interaction between α1β2^{HA}γ2 (wt,wt), α1β2^{HA,PL}γ2 (β2 – P252L,P252L) and α1^{PL}β2^{HA,PL}γ2^{PL} (5*PL mutation) receptor combinations ($F_{(4,18)}$ = 0.28; P = 0.88) but significant differences between receptor type and cDNA quantities ($F_{(2,18)}$ = 59 and $F_{(2,18)}$ = 11; $P < 0.0001$ and P = 0.0009, respectively). Right, a two-way ANOVA revealed no significant interaction between receptor combinations ($F_{(4,18)}$ = 0.35; P = 0.84) but significant differences between receptor type and cDNA quantities ($F_{(2,18)}$ = 70 and $F_{(2,18)}$ = 5.5; $P < 0.0001$ and P = 0.014, respectively). Indicated significance levels represent Dunnett's multiple comparison test with *: $P < 0.05$.

SI4. Maximal open probability estimation of wildtype and M1 proline mutated α1β2/3γ2 GABAARs.

To investigate for variant-induced changes in GABA gating efficiency, the maximal open probability was estimated using a pharmacological methodology. Etomidate is a highly efficacious allosteric modulator of $GABA_ARs$ and by co-applying a saturating concentration of etomidate with a maximally efficacious concentration of GABA, as many receptors as possible will enter the open state (30, 40, 51, 52). Under the assumption that receptors exposed to GABA and etomidate have an open probability approaching 1, the maximum open probability of GABA alone can be calculated. The Est. $P_{O,max}$ values of wildtype α1β2γ2 and α1β3γ2 receptors were 0.85 and 0.75, respectively, in good agreement with previous observations (Figure SI4) (34, 40). Double-mutant receptors containing M1 proline mutations in both α1, β2 or β3 subunits displayed Est. $P_{O,max}$ values of 0.63-0.92. An One-way ANOVA test revealed no difference in Est. $P_{O,max}$ values between any receptor combinations. Hence, the M1 proline mutations do not appear to affect the gating efficiency of GABA.

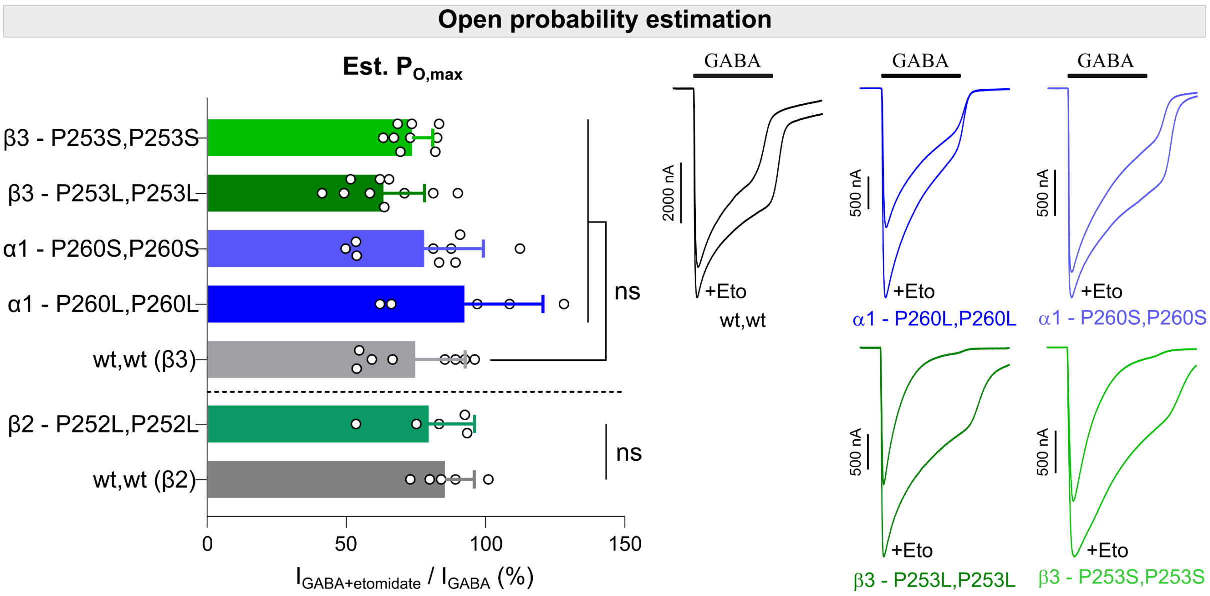

Figure SI4. Estimated maximal open probability of M1 proline mutated $GABA_A$ receptors. To estimate the maximal open probability of wildtype (wt) α1β2/3γ2 receptors and double-mutant receptors containing M1 proline mutations, peak current amplitudes evoked by a maximally efficacious concentration of GABA (10 mM) was compared with amplitudes evoked by GABA (10 mM) + Etomidate (Eto, 10 μM). Left, mean values ± SD of n = 5-10 individual experiments with individual values indicated. An One-way ANOVA revealed no significant differences between the estimated $P_{O,max}$ values (Kruskal-Wallis test, P = 0.14). Right, bars above the representative traces indicate the 30-s application time of GABA or GABA+Eto.

SI5. RMSD of the α1β2γ2 $GABA_A$ receptor Cα atoms.

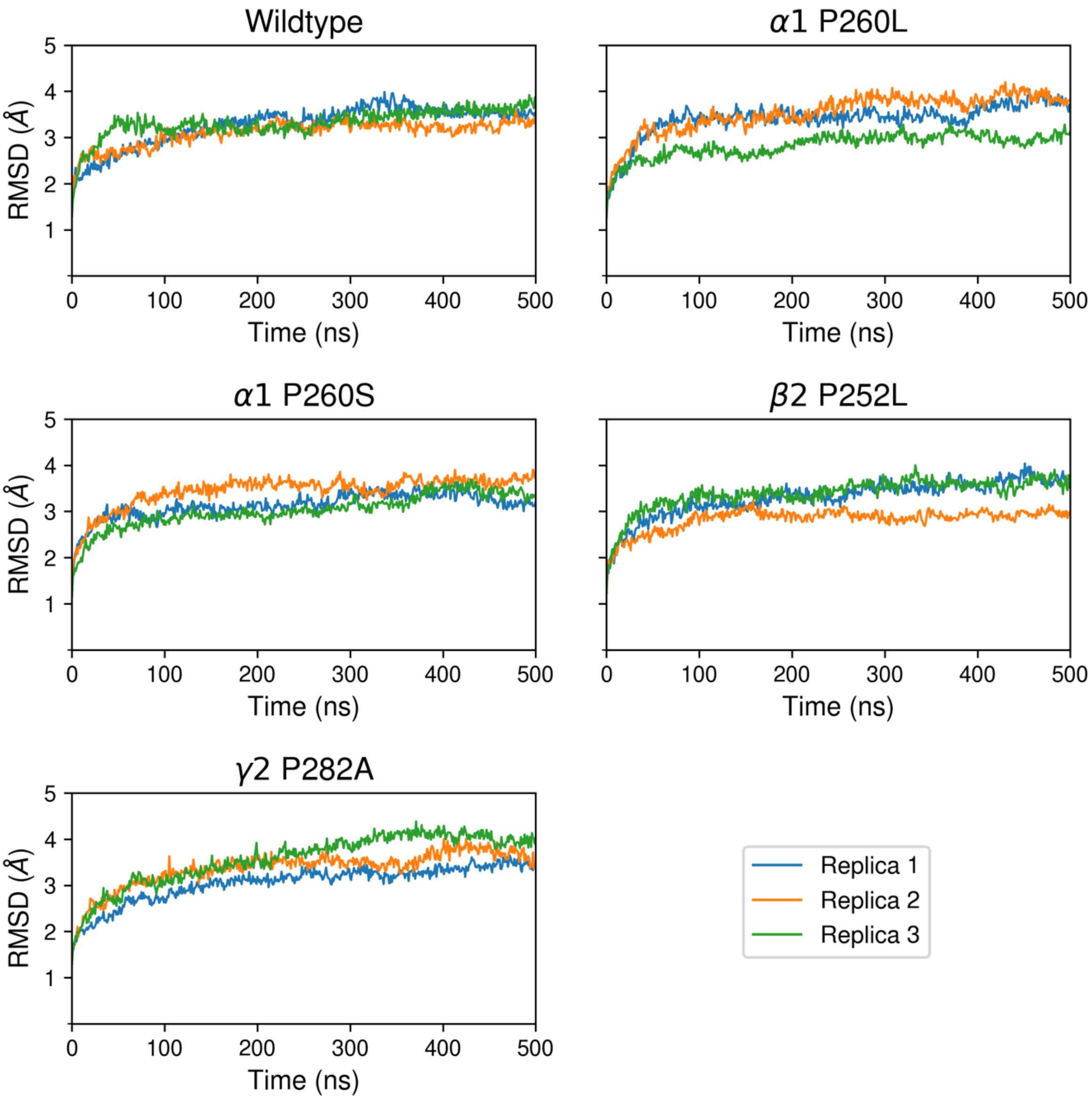

Figure SI5: Root mean squared deviation (RMSD) of the α1β2γ2 $GABA_A$ receptor Cα atoms over time with and without M1 proline mutants. RMSD was calculated every 1 ns following superimposition of the protein Cα atoms on the starting structure (PDB: 6X3Z) (42). The first, second and third simulation replicas are coloured in blue, orange, and green, respectively.

SI6. Pore radius profile of the α1β2γ2 GABA$_A$ receptor.

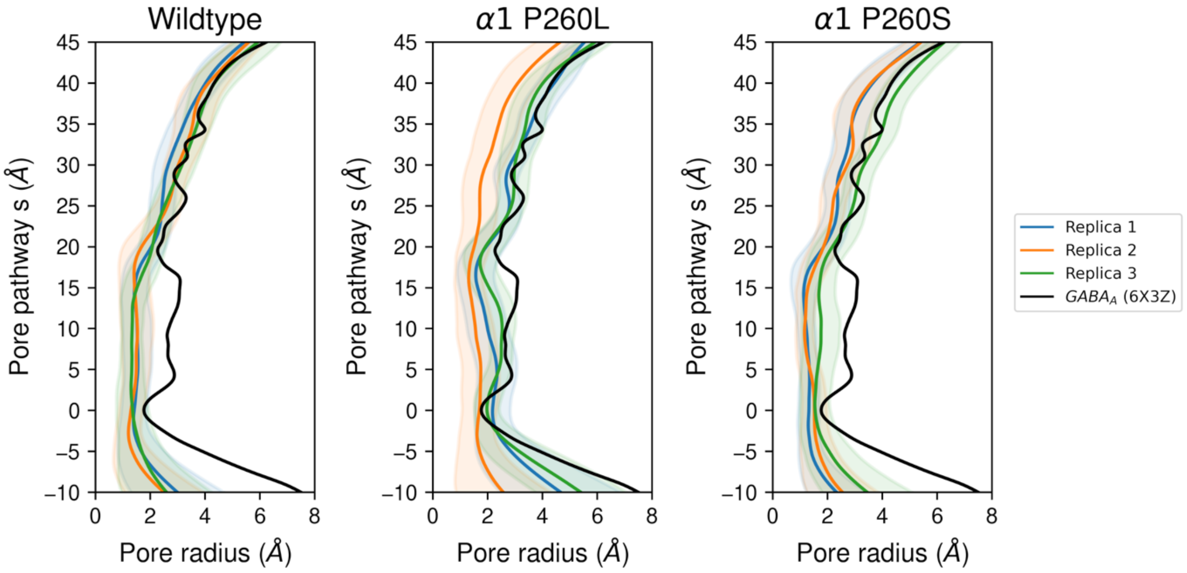

Figure SI6: Pore radius profile of the α1β2γ2 GABA$_A$ receptor with wt sequence, α1 P260L or P260S mutations. The pore radius was averaged over the last 300 ns of triplicate simulations, solid blue (replica 1), orange (replica 2) and green (replica 3) lines represent the mean, and the faded outlines represent SD. The black line represents the profile of the α1β2γ2 GABA$_A$ receptor structure (PDB: 6X3Z) in a desensitized conformation. Along the pore pathway, the -2' gate (α1 P280, β2 A272 and γ2 P302) is positioned at ~ 0 Å and the 9' gate (α1 L291, β2 L283 and γ2 L313) is positioned at ~ 15 Å.

SI7. Secondary structure (SS) propensity for M1 helix residues.

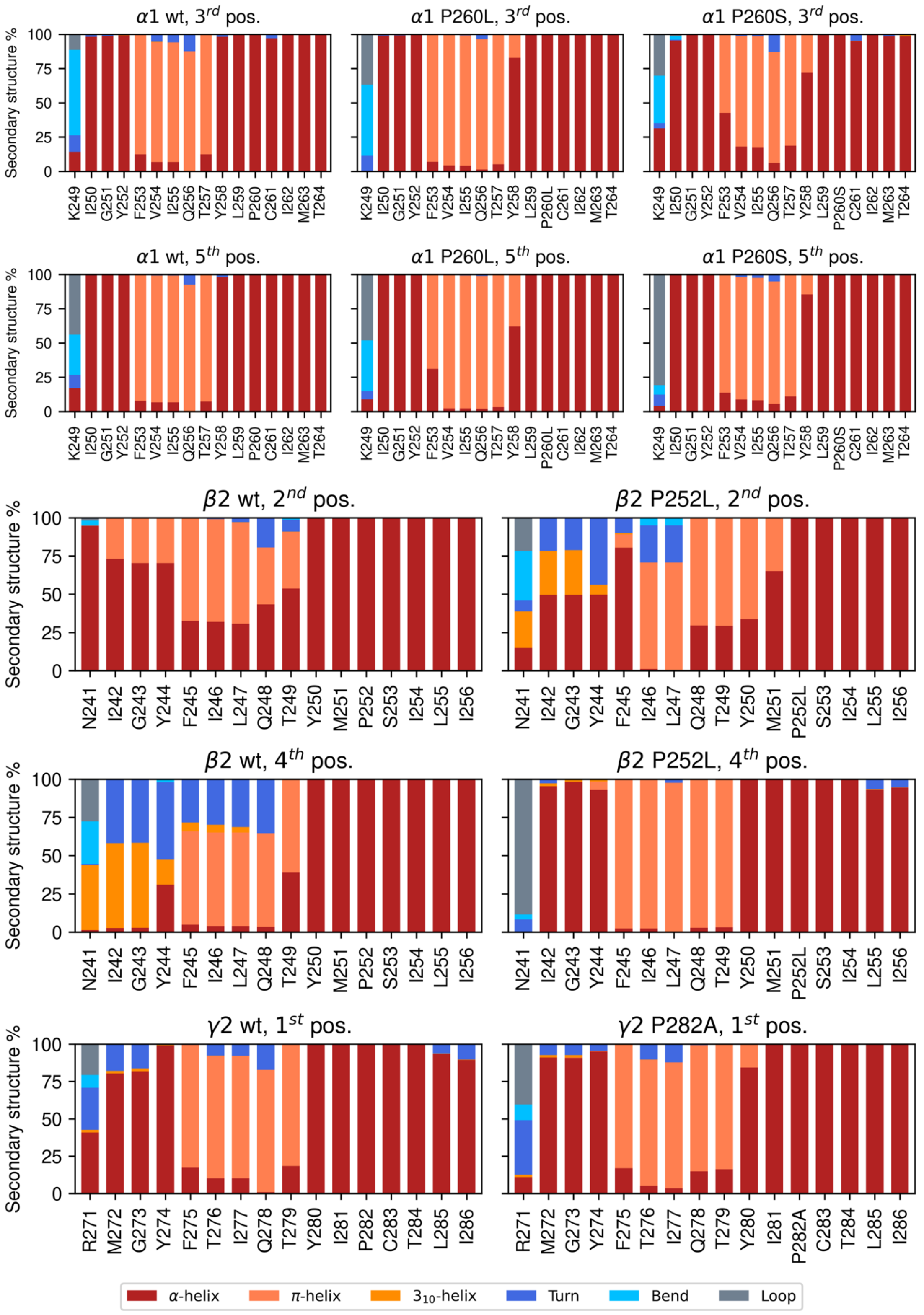

Figure SI7: Secondary structure (SS) propensity for M1 helix residues of the α1β2γ2 GABA$_A$ receptor with and without proline mutants. The values for each residue are averaged from the last 300 ns of triplicate molecular dynamics simulations. The SS propensities represent the % of simulation frames where a particular secondary structure was observed: α-helix (maroon), π-helix (salmon), 3$_{10}$-helix (orange), turn (dark blue), bend (lightblue) and unstructured loop (gray).

SI8. H-bond analysis of the M1 helix backbone of the α1 subunit, 3rd position.

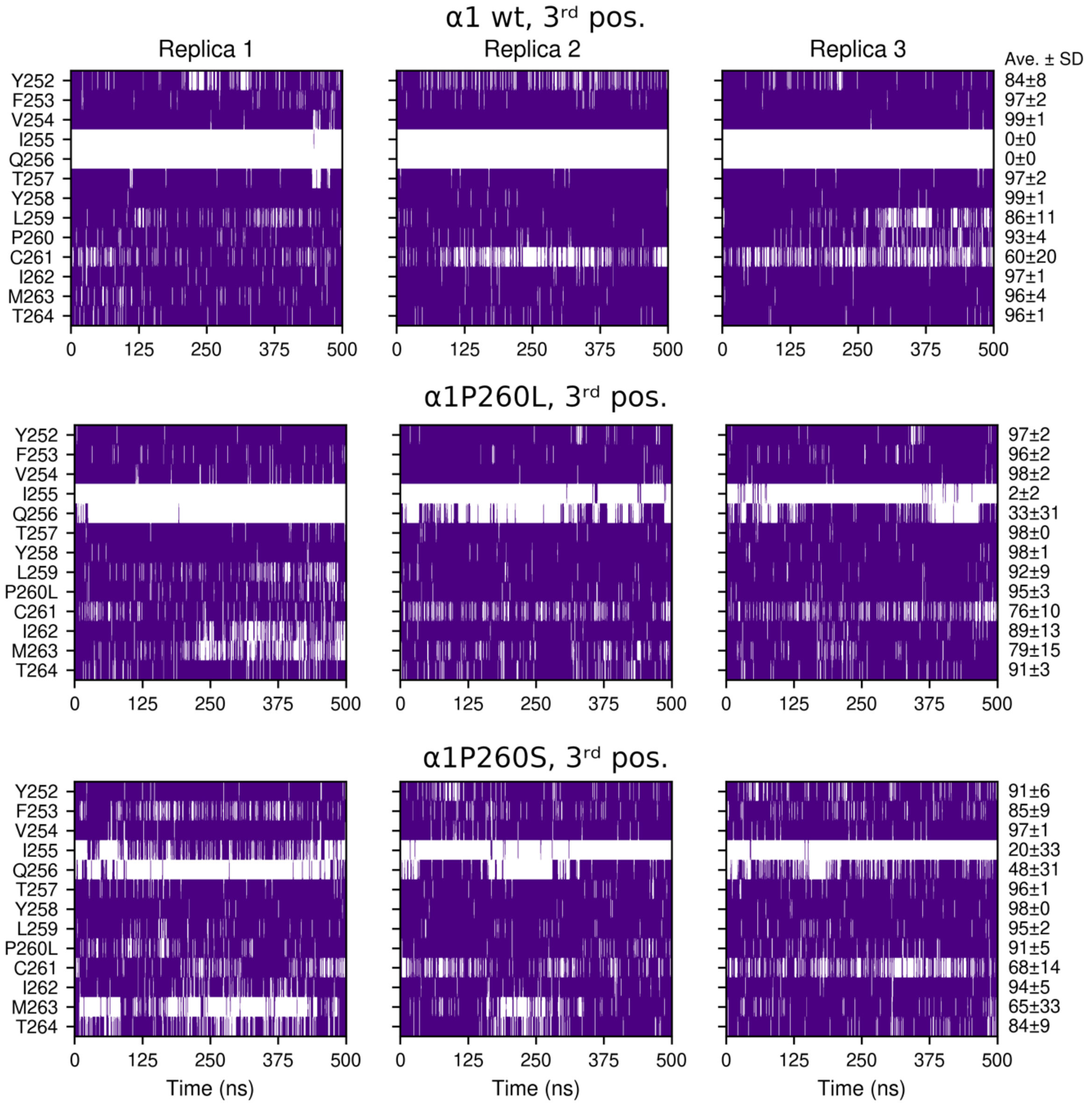

Figure SI8: H-bond analysis of the M1 helix backbone of the α1 subunit, 3rd position with wt sequence, α1 P260L or P260S mutations. Purple dashes on the plots indicate the presence of a H-bond from the backbone carbonyl oxygen to NH atoms below it. The presence of a H-bond was calculated every 1 ns of the simulation, the left, middle and right panels represent the three simulation replicas. The values to the right of each plot represent mean ± SD across the three simulation replicas.

SI9. H-bond analysis of the M1 helix backbone of the α1 subunit, 5th position.

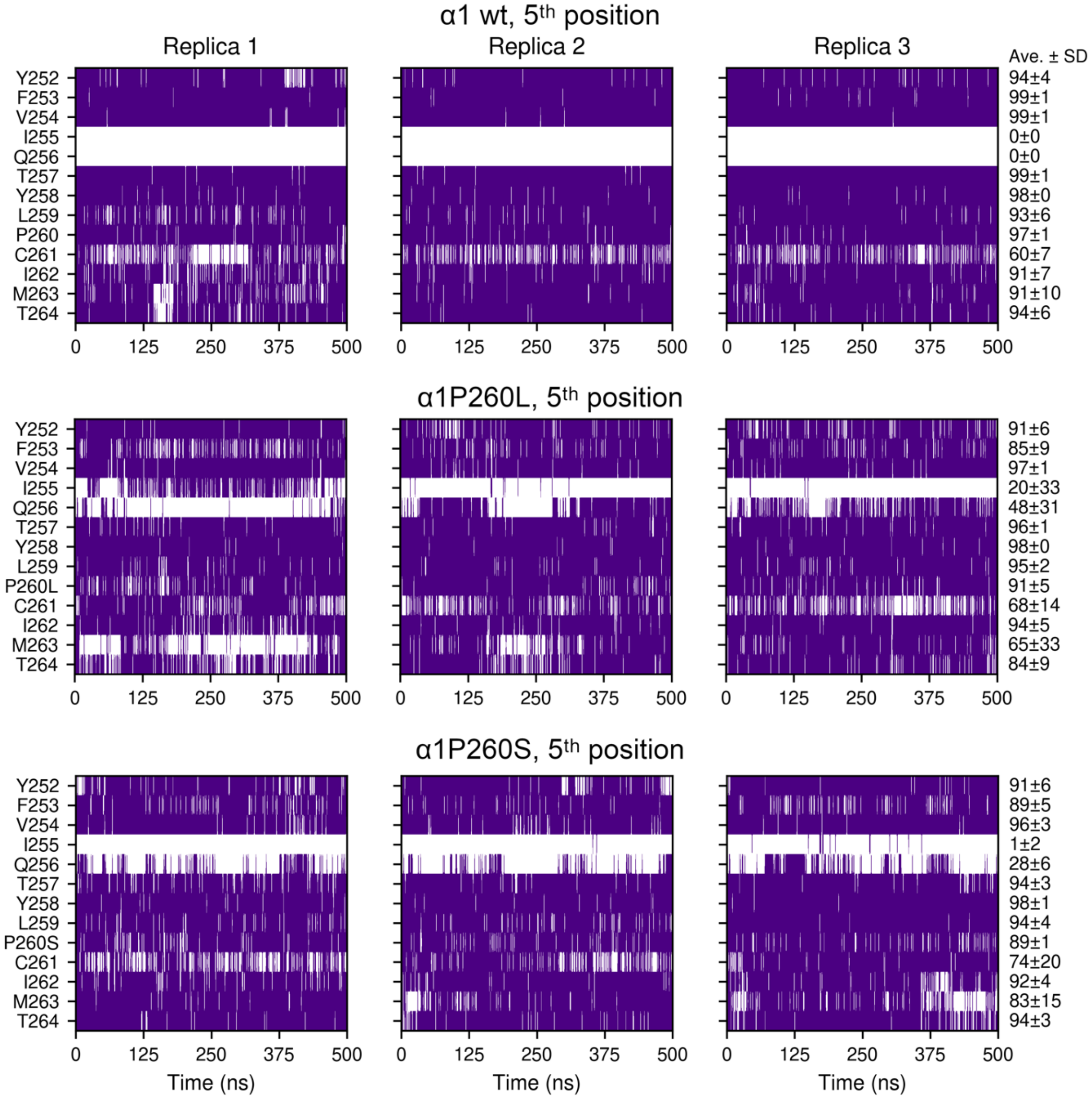

Figure SI9: H-bond analysis of the M1 helix backbone of the α1 subunit, 5th position with wt sequence, α1 P260L or P260S mutations. See Figure SI8 for details about the plots.

SI10. H-bond analysis of the M1 helix backbone of the β2 subunit.

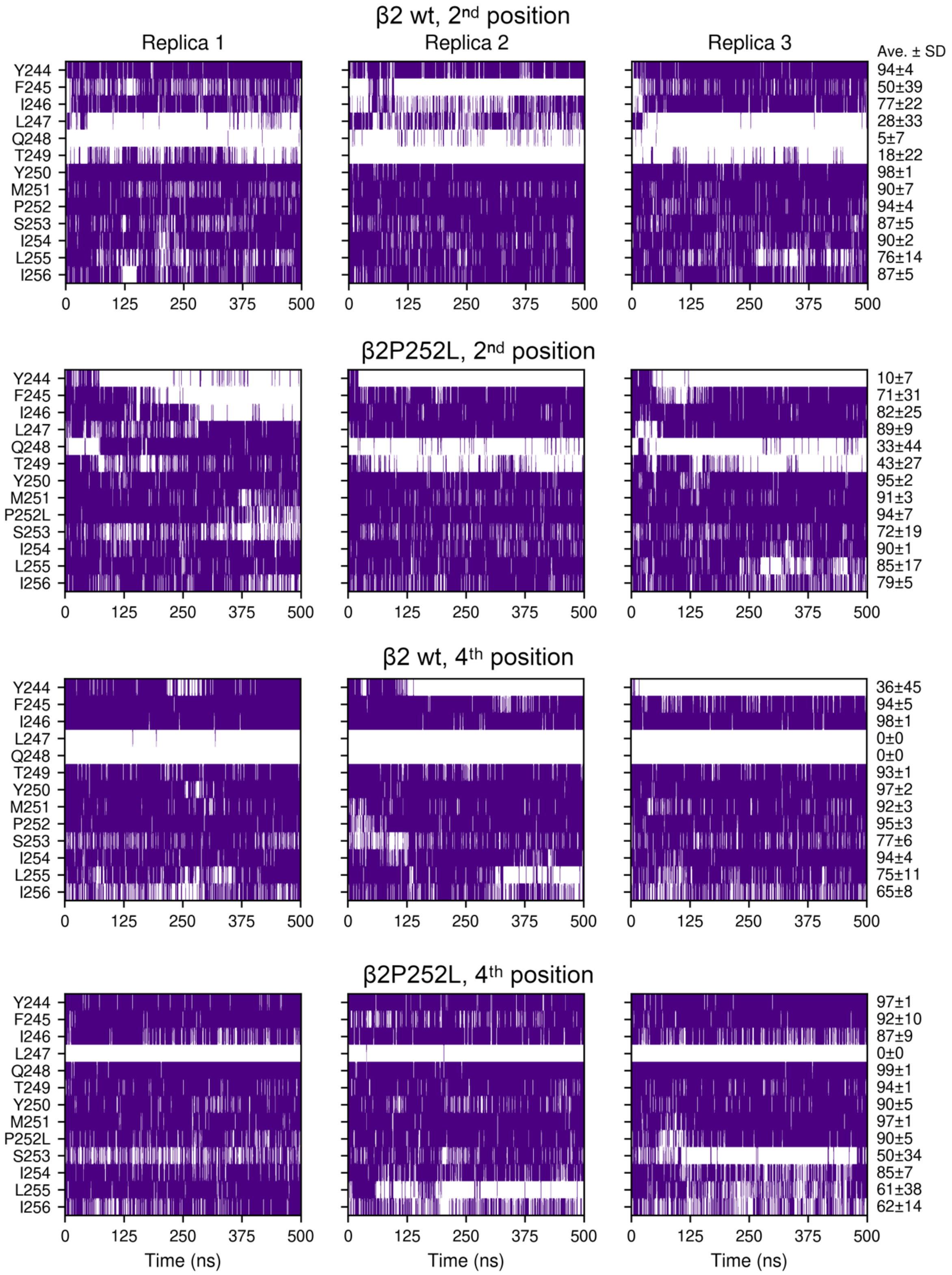

Figure SI10: H-bond analysis of the M1 helix backbone of the β2 subunit, 2nd and 4th positions with wt sequence or the β2 P252L mutation. See Figure SI8 for details about the plots.

SI11. H-bond analysis of the M1 helix backbone of the γ2 subunit.

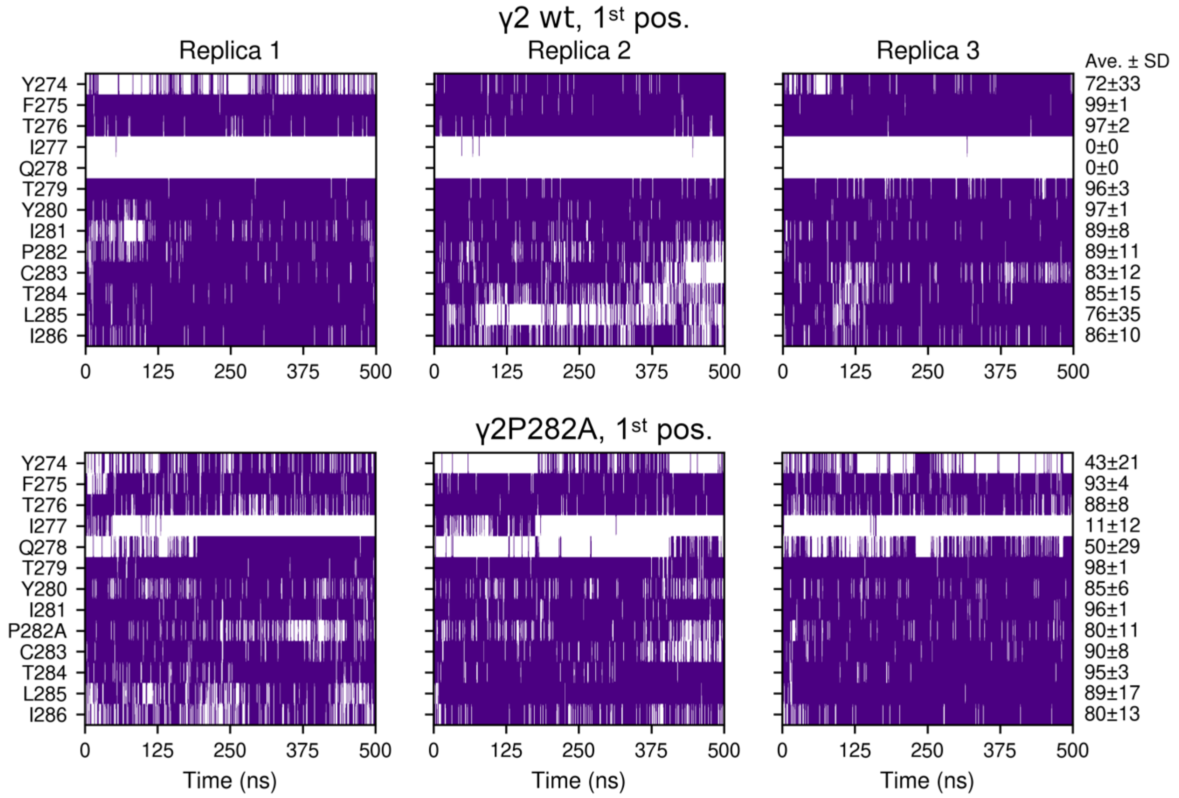

Figure SI11: H-bond analysis of the M1 helix backbone of the γ2 subunit, 1st position with wt sequence or the γ2 P282L mutation. See Figure SI8 for details about the plots.

SI12. Curvature profile of the M1 helix tip.

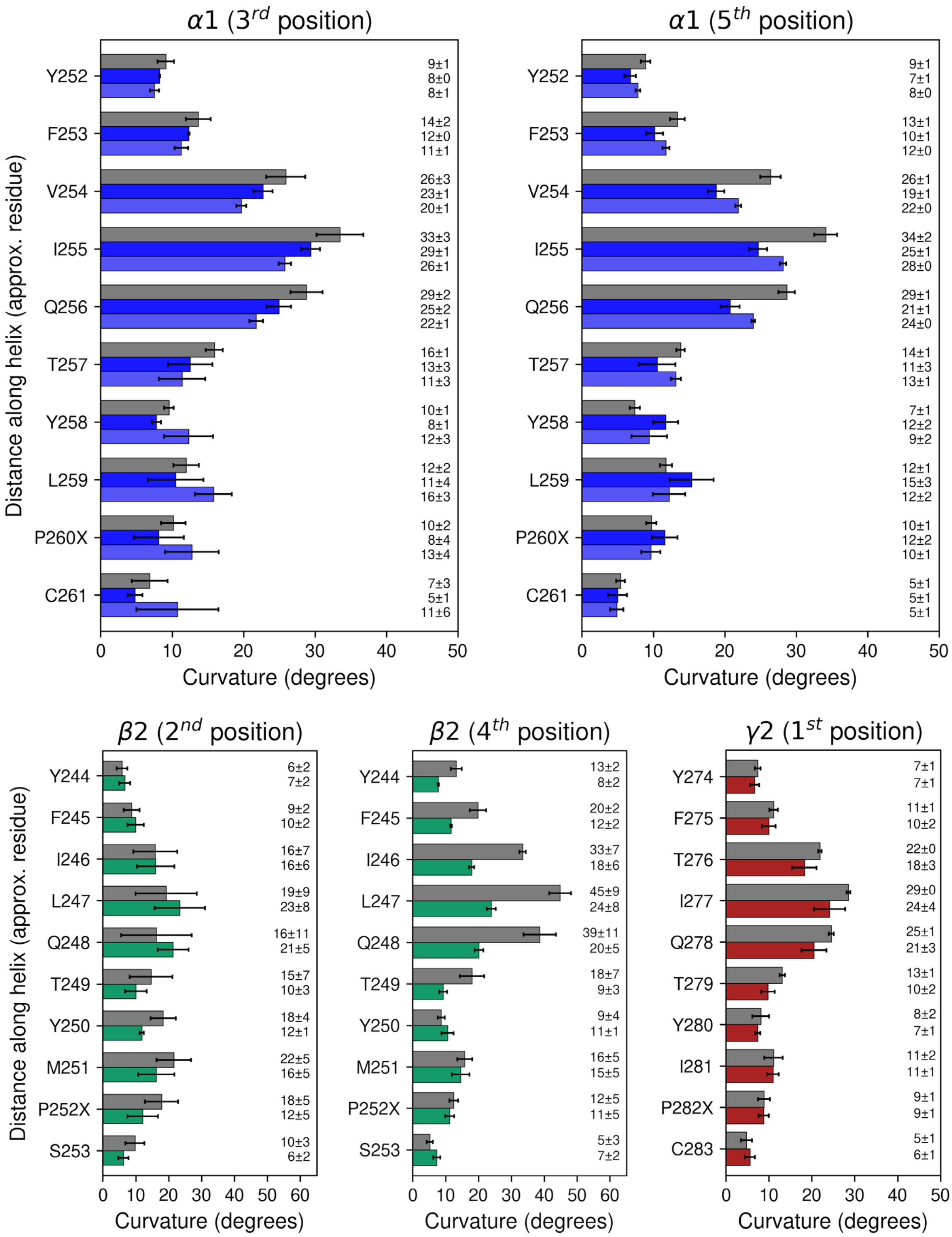

Figure SI12: Curvature profile of the M1 helix tip in the α1β2γ2 GABA$_A$ receptor. Per residue helix curvature is calculated using the Bendix tool from the last 300 ns of triplicate MD simulations. The average curvature from each of the three replicas is used to calculate overall mean ± SD. The suffix X after the M1 proline in each plot indicates the presence or absence of a mutation. Bar colouring as follows: wt (grey) α1 P260L (blue), α1 P260S (light blue), β2 P252L (green) and γ2 P282A (red) mutants.

SI13. GABA$_A$R subunit sequence homology and recommendations when comparing paralogous variants

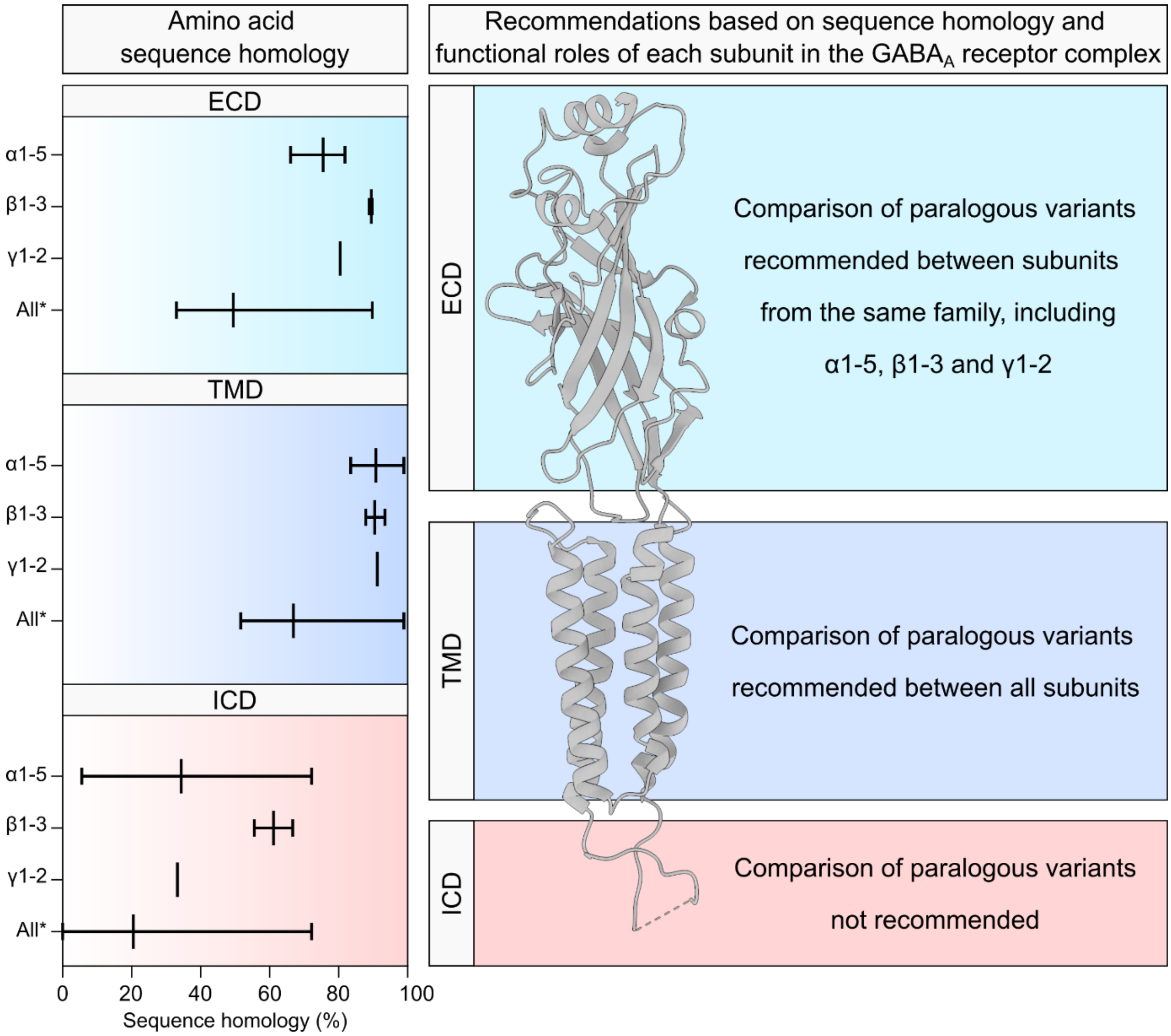

Figure SI13. Recommendations when using known variants with functional data to predict novel paralogous variants. Left: Amino acid sequence homology of the major GABA$_A$R subunits (α1-5, β1-3, γ1-2 and δ) were computed using Clustal Omega alignment tool on MegAlign Pro (DNASTAR, USA) and presented as mean and range (%). Sequence homology between subunits from the same family was calculated for α, β and γ subunits, and an overall homology comparison was performed between all eleven subunits (All*). **Right**: Based on sequence homology data and the functional roles of each GABA$_A$R subunit in the receptor complex, we have summarised our recommendations when comparing paralogous variants between subunits for each of the three main subunit domains. ECD: extracellular domain; TMD: transmembrane domain; ICD: intracellular domain.

SI14. Materials and Methods extended version

Molecular biology

Concatenated γ2-β2-α1-β2-α1 and γ2-β3-α1-β3-α1 receptors were created as previously described (29, 30). To create single mutant-containing receptors, mutated subunits were purchased from Genscript (Singapore) and subcloned into tetramer constructs lacking the subunit to be cloned in by use of a standardised restriction digest and ligation protocol. Double variant-containing receptors (in the case of α1, β2 and β3 variants) were created accordingly, by subcloning of the second mutant subunit into a pentameric single variant construct, i.e., replacing one of the wildtype subunits in this construct. To confirm successful cloning, all constructs were subjected to Sanger sequencing (Australian Genome Research Facility, Westmead, New South Wales) and diagnostic restriction digestion. To generate cRNA for microinjection, the cDNA constructs were linearised for *in vitro* transcription to synthesise cRNA using the mMessage mMachine T7 Transcription Kit (Thermo Fisher) and stored at -20 ºC.

Xenopus laevis oocytes and electrophysiology

Xenopus laevis oocytes were obtained from the Victor Chang Cardiac Research Institute, Darlinghurst, New South Wales. Oocytes from stage V or VI were microinjected with ~25 ng of cRNA and incubated for at least 48 h at 18 ºC for adequate receptor expression. On the day of recording, the oocytes were impaled with borosilicate glass microelectrodes filled with 3 M KCl and with a resistance between 0.2 to 2 MΩ. The cells were then voltage clamped at -50 mV using an Axon GeneClamp 500B amplifier (Molecular Devices). Amplified currents were sampled at 200 Hz (10 kHz for desensitisation experiments), filtered at 10 Hz by a low-pass Bessel filter (Axon GeneClamp 500B) and digitalised with Digidata 1440A (Molecular Devices) before being analysed on pClamp 10.2 suite (Molecular Devices) or LabChart reader version 8.1 (AD Instruments). All electrophysiological experiments were conducted in a minimum of two batches of oocytes with wildtype controls running along the mutant receptor recording on each experimental day to account for variations between the batches.

GABA concentration-response and maximal GABA-evoked current amplitudes

To obtain GABA concentration-response relationships displayed by wildtype and variant-containing receptors, increasing GABA concentrations (9-10 concentrations) were applied to the oocytes for 30 s at a rate of 2 mL/min via a custom-built solution delivery system that allows for rapid solution exchange. Throughout the experiment, the oocytes were continuously perfused with ND96 (96 mM NaCl, 2 mM KCl, 1 mM $MgCl_2$, 1.8 mM $CaCl_2$ and 10 mM HEPES; pH 7.4). The Hill equation was fitted to peak GABA-evoked current amplitudes for individual oocytes to determine the EC_{50}:

$$I = Abs.I_{max}([GABA]^{nH}/([GABA]^{nH} + [EC_{50}]^{nH}))$$

$Abs.I_{max}$ is the absolute maximum current, EC_{50} is the GABA concentration required to elicit half-maximum current response, [GABA] refer to GABA concentration, and nH is the Hill slope. The mean EC_{50} was calculated for both the wildtype ($EC_{50,wt}$) and variants (EC_{50}), and subsequently transformed into $LogEC_{50}$ (i.e., $LogEC_{50,wt}$ and $LogEC_{50}$, respectively). The difference in $LogEC_{50}$ between the wildtype and mutant constructs ($\Delta LogEC_{50}$) for fully parallel experiments performed on the same experimental day were then calculated to determine the shift in GABA potency:

$$\Delta\, Log\, EC_{50} = Log\, EC_{50,wt} - \; Log\, EC_{50}$$

Mean $\Delta LogEC_{50}$ was calculated for all mutants and presented as mean ± S.D.

To determine the maximum current amplitudes (I_{max}) evoked through the wildtype and variant-containing receptors, a saturating 10 mM concentration of GABA was applied to the oocytes to elicit peak currents at wildtype and mutant receptors. The I_{max} for fully parallel experiments performed on the same experimental day were then calculated:

$$I_{max} = \frac{Abs.I_{max}}{Abs.I_{max,wt}}$$

($Abs.I_{max}$) is the absolute maximum current and ($Abs.I_{max,wt}$) is the daily average of wildtype experiments.

Well-established assays

The GABA sensitivity and maximum GABA-evoked current amplitude assays have been used in previous studies with large cohorts of $GABA_AR$ variants (3-5). In line with the recommendations from Brnich, Abou Tayoun (41), both parameters qualify as well-established assays that: (i) define the disease mechanism in LOF and GOF variants; (ii) model the disease mechanism where changes in GABA sensitivity are associated with LOF or GOF; (iii) include known pathogenic LOF, GOF, and benign variants; and (iv) normalise EC_{50} and I_{max} values to wildtype controls run on each recording day.

Desensitisation protocol

Two elements of desensitisation properties were evaluated: the initial current decay rate (k) and steady-state currents as a ratio to peak current (I_{ss}/I_{pk}). Experiments were performed in three groups run in parallel with statistical comparisons between variant receptors and wildtype controls recorded on the same experimental day. The desensitisation experiments followed the protocol outlined in Lin, Ahring (34) with some variations. Briefly, the oocytes were perfused with ND96 via a semi-automatic gravity-driven perfusion system at 1 mL/s for 4 min to stabilise membrane conductance. Next, 3 mM GABA was applied to the oocytes for 150 s to record current decay activity. To obtain the decay rate constant, non-linear regression was performed on the current traces using GraphPad Prism 10.0.1 and fitted using the following one-phase exponential decay equation:

$$Y = (Y_0 - Plateau) \cdot e^{-kx} + Plateau$$

where Y represents current amplitude in nA and x being the time. To derive I_{ss}/I_{pk} from the recordings, the steady state (Plateau) current (I_{ss}) was divided against the peak current (I_{pk}) elicited for each experiment.

Enzyme-linked immunosorbent assay (ELISA)

The construction of the (human) α1-pcDNA3.1, β2-pcDNA3.1 and γ2- pcDNA3.1 plasmids has been described previously (53). Hemagglutinin (HA) epitope-tagged versions of α1 and β2 were constructed using the splicing by overlap extension PCR technique by insertion of the nucleotide sequence for the HA epitope (Tyr-Pro-Tyr-Asp-Val-Pro-Asp-Tyr-Ala) between the nucleotide sequences of the signal peptide and the mature subunit protein (α1: …Ser-Tyr-Gly-HA-Gln-Pro-Ser…; β2: …Val-Cys-Ala-HA-Gln-Ser-Val…). M1 proline-to-leucine mutations were introduced into wildtype α1, β2 and γ2 subunits as well as HA epitope-tagged $\alpha1^{HA}$ and $\beta2^{HA}$ subunits by use of the QuikChange mutagenesis kit (Stratagene). The validity and absence of unwanted mutations in the constructed cDNAs was verified by DNA sequencing (Eurofins Genomics, Ebersberg, Germany).

Cell surface expression levels of HA-tagged α1 and β2 ($\alpha1^{HA}$ and $\beta2^{HA}$) subunits and M1 proline-to-leucine mutants (PL) of these ($\alpha1^{HA,PL}$ and $\beta2^{HA,PL}$) transiently expressed together with the respective supplementary untagged wildtype and mutant PL subunits needed for α1β2γ2 receptor assembly in tsA201 cells were quantified in an ELISA. To evaluate the additive effect of introducing additional PL mutated subunits on cell surface expression levels, the expression properties of α1β2γ2 receptors containing PL mutations in all five subunits were also characterised. The ELISA was performed on cells expressing different combinations of extracellularly HA-tagged versions of wildtype α1 or β2 ($\alpha1^{HA}$ or $\beta2^{HA}$), the PL mutants of these ($\alpha1^{HA,PL}$ or $\beta2^{HA,PL}$) and the supplementary untagged wildtype or PL-mutated α1, β2 and γ2 subunits, and the cell surface and total expression levels of these tagged subunits were determined in intact and permeabilised cells, respectively.

The tsA201 cells were cultured in GlutaMAX-I Dulbecco's Modified Eagle's Medium (Invitrogen) supplemented with 10% fetal bovine serum, penicillin (100 U/ml) and streptomycin (100 µg/ml) in a humidified atmosphere of 5% CO_2 at 37 °C. Exponentially growing cells were seeded into 6 cm tissue culture dishes (1×10^6 cells/dish) and transfected 20-24 h later with a total of 4.0 µg cDNA using PolyFect (Qiagen) according to the manufacturer's protocol.

Transfections for each receptor combination were done using three different cDNA mixtures to establish cell surface expression levels spanning the dynamic range of the ELISA assay. These contained α1 + β2 + γ2: 0.33 µg + 0.33 µg + 0.33 µg or 0.11 µg + 0.11 µg + 0.11 µg or 0.037 µg + 0.037 µg + 0.037 µg supplemented with 3.0 µg, 3.67 µg and 3.89 µg "empty" pcDNA3.1, respectively, yielding a total of 4.0 µg cDNA/6-cm dish. Parallel transfections of cells with "empty" pcDNA3.1 (4.0

μg cDNA/6-cm dish) were used as "mock" controls to assess non-specific binding of the antibody, and transfections with cDNA mixtures of 1.33 μg + 1.33 μg + 1.33 μg of $\alpha1^{HA}$ + β2 + γ2 or α1 + $\beta2^{HA}$ + γ2 were used as normalisation reference for the $\alpha1^{HA}$- and $\beta2^{HA}$-experiments, respectively.

16-18 h after the transfection, the cells were plated into PDL-coated 48-well plates (1×10^5 cells/well). The ELISA experiment was initiated 24-25 h later, and it was performed essentially as previously described (54). The cells were washed two times with ice-cold wash buffer (phosphate-buffered saline supplemented with 1 mM $CaCl_2$) and incubated with an ice-cold 4% paraformaldehyde solution for 12 min on ice, after which the following steps were performed at room temperature. The cells were washed three times with wash buffer, incubated for 30 min in blocking solution (3% skim milk powder in 50 mM Tris-HCl, 1 mM $CaCl_2$, pH 7.5), and incubated with rat monoclonal anti-HA-peroxidase conjugated antibody (clone 3F10, Sigma-Aldrich, diluted 1:1000 in blocking solution) for 1 h. The expression of the HA-tagged proteins was determined in parallel triplicate wells, where the blocking and antibody incubation steps were performed in the absence (intact cells: cell surface expression) or in the presence of 0.1% Triton X-100 (permeabilised cells: whole cell expression). Cells were then washed three times with wash buffer, and expression of the HA-tagged proteins was quantified using 3,3′,5,5′-tetramethylbenzidine liquid substrate system (Sigma Aldrich) and H_2SO_4. The absorbance of the supernatants was determined at 450 nm. The average absorbance in the wells with "mock"-transfected tsA201 cells were subtracted from the absorbance measured in all other wells, and the resulting absorbance reflecting specific expression of the HA-tagged proteins were normalised to the cell surface expression measured in wells with tsA201 cells transfected with $\alpha1^{HA}$ + β2 + γ2 or α1 + $\beta2^{HA}$ + γ2 (both 1.33 μg + 1.33 μg + 1.33 μg). All experiments were performed in triplicate, and data are based on three independent experiments.

Molecular dynamics simulations

The α1β2γ2 $GABA_AR$ structure (PDB 6X3Z) (42) served as the initial conformation for molecular dynamics simulations. Copies of the protein structure were made with the M1 proline replaced by mutant residues α1 P260L, α1 P260S, β2 P252L, and γ2 P282A such that five separate structures including wildtype were used. The CHARMM-GUI webserver (43) was used to prepare hexagonal simulation cells for each of the protein structures. In brief, the proteins were placed in a 1-palmitoyl-2-oleoyl-sn-glycero-3-phosphocholine (POPC) lipid bilayer with 204 and 212 lipids in the top and bottom membrane leaflets, respectively. The proteins were immersed in ~48,000 water molecules together with 0.15 M NaCl including excess of Cl^- ions to neutralise overall charge. The systems were parameterised using the GROMOS 54A7 force field (44), united atom parameters for GABA were created using the using the ATB web server (45), simulations were performed using GROMACS 2021.4 (46). The systems were energy minimized using the steepest descent algorithm and equilibrated in five sequential 1 ns simulations with decreasing positional restraints on the protein and ligands (1000 kJ mol^{-1} nm^{-1},

500 kJ mol^{-1} nm^{-1}, 100 kJ mol^{-1} nm^{-1}, 50 kJ mol^{-1} nm^{-1}, and 10 kJ mol^{-1} nm^{-1}). The protein backbone and ligand atoms were restrained at 10 kJ mol^{-1} nm^{-1} for an additional 10 ns before completely lifting restraints. Following equilibration, each system was simulated in triplicate for 500 ns with a 2 fs timestep. The Berendsen barostat was used to main pressure at 1 bar with semi-isotropic coupling (τ_P=0.5 ps and isothermal compressibility=4.5x10^{-5} bar). The Bussi-Donadio-Parrinello velocity rescale thermostat was used to maintain temperature at 300 K. The LINCS algorithm was to constrain the covalent bond lengths of the solute and the SETTLE algorithm was used to constrain the geometry of water molecules.

All simulation analyses were conducted on simulation frames spaced 1 ns apart from each replica. The first 200 ns of each production simulation was omitted as equilibration for pore radius profile, secondary structure propensity and helix curvature analyses. Root mean squared deviation (RMSD) and secondary structure propensity analyses were performed using the MDTraj library (47). The RMSD of protein Cα atoms was calculated relative to the initial protein conformation after least squares fitting of the trajectory to the initial conformation. H-bond analysis was done to calculate the propensity for H-bonds to form between backbone carbonyl oxygens of the M1 helix residues to NH atoms below them, this was conducted using the PROLIF tool (48). The pore radius profile over time was calculated using the CHAP tool with default settings (49). The M1 helix curvature over time was calculated using the Bendix tool with default settings (50). The electrostatic interactions were calculated using the Particle Mesh Ewald summation, and non-covalent interactions were determined via the Verlet scheme with a 1.0 nm cut-off, periodic boundary conditions were implemented.
